# Pharmacological characterisation of S 47445, a novel positive allosteric modulator of AMPA receptors

**DOI:** 10.1371/journal.pone.0184429

**Published:** 2017-09-08

**Authors:** Sylvie Bretin, Caroline Louis, Laure Seguin, Stéphanie Wagner, Jean-Yves Thomas, Sylvie Challal, Nathalie Rogez, Karine Albinet, Fabrice Iop, Nadège Villain, Sonia Bertrand, Ali Krazem, Daniel Bérachochéa, Stéphanie Billiald, Charles Tordjman, Alex Cordi, Daniel Bertrand, Pierre Lestage, Laurence Danober

**Affiliations:** 1 Pôle Innovation Thérapeutique Neuropsychiatrie, Institut de Recherches Internationales Servier, Suresnes, France; 2 Pôle Innovation Thérapeutique Neuropsychiatrie, Institut de Recherches Servier, Croissy sur Seine, France; 3 Neurofit, Illkirch-Graffenstaden, France; 4 HiQScreen, Vésenaz Geneva, Switzerland; 5 Centre de Neurosciences Intégratives et Cognitives, Université Bordeaux 1, Talence, France; 6 Pôle Expertise Recherche et Biopharmacie, Institut de Recherches Servier, Suresnes, France; Bilkent University, TURKEY

## Abstract

S 47445 is a novel positive allosteric modulator of alpha-amino-3-hydroxy-5-methyl-4-isoxazole-propionic acid (AMPA) receptors (AMPA-PAM). S 47445 enhanced glutamate’s action at AMPA receptors on human and rat receptors and was inactive at NMDA and kainate receptors. Potentiation did not differ among the different AMPA receptors subtypes (GluA1/2/4 flip and flop variants) (EC_50_ between 2.5–5.4 μM), except a higher EC_50_ value for GluA4 flop (0.7 μM) and a greater amount of potentiation on GluA1 flop. A low concentration of S 47445 (0.1 μM) decreased receptor response decay time of GluA1flop/GluA2flip AMPA receptors and increased the sensitivity to glutamate. Furthermore, S 47445 (0.1 and 0.3 μM) in presence of repetitive glutamate pulses induced a progressive potentiation of the glutamate-evoked currents from the second pulse of glutamate confirming a rapid-enhancing effect of S 47445 at low concentrations. The potentiating effect of S 47445 (1 μM) was concentration-dependently reversed by the selective AMPA receptor antagonist GYKI52466 demonstrating the selective modulatory effect of S 47445 on AMPA receptors. Using an AMPA-kainate chimera approach, it was confirmed that S 47445 binds to the common binding pocket of AMPA-PAMs. S 47445 did not demonstrate neurotoxic effect against glutamate-mediated excitotoxicity *in vitro*, in contrast significantly protected rat cortical neurons at 10 μM. S 47445 was shown to improve both episodic and spatial working memory in adult rodents at 0.3 mg/kg, as measured in the natural forgetting condition of object recognition and T-maze tasks. Finally, no deleterious effect on spontaneous locomotion and general behavior was observed up to 1000 mg/kg of S 47445 given acutely in rodents, neither occurrence of convulsion or tremors. Collectively, these results indicate that S 47445 is a potent and selective AMPA-PAM presenting procognitive and potential neuroprotective properties. This drug is currently evaluated in clinical phase 2 studies in Alzheimer’s disease and in Major Depressive Disorder.

## Introduction

Ionotropic glutamate AMPA (α-amino-3-hydroxy-5-methyl-4-isoxazole propionic acid), NMDA (N-methyl-D-aspartate) and kainate receptors are cation-permeable channels responsible for excitatory synaptic transmission in the mammalian brain, with distinct contribution to components of the synaptic excitatory signals [1–3]. The major role of AMPA receptors in synaptic plasticity and memory processes has been widely discussed due to rapid kinetics and trafficking to synapses [4–6]. AMPA receptor ligand binding cores form dimeric units in a tetrameric assembly [7, 8] composed of heteromeric or homomeric combinations of four different subunit proteins (GluA1-4) [2, 4]. The presence of GluA2 subunit results in reduced calcium permeability. Additionally, each subunit undergoes alternative splicing and can be expressed as a flip or flop splice variants [9, 10] with distinct pharmacological properties. Moreover, flip splice variants desensitize about four times slower than flop variants [11, 12]. AMPA receptors have a modular structure with four domains in each subunit: a large extracellular N-terminal domain (NTD), a ligand-binding domain (LBD), the ion channel and an intracellular C-terminal domain determinant in the synaptic targeting. A large body of evidence suggests that the extracellular portion of the AMPA receptor composed of the LBD and NTD mediates complex regulation in gating mechanisms of the receptors and in kinetics and strength of glutamatergic synaptic transmission [1, 8].

Interestingly, structural and functional studies have shown that positive allosteric modulators of AMPA receptors (AMPA-PAMs) identified so far bind to a common allosteric binding pocket of LBD dimers located at the interface between two subunits [13–18]. Binding of an AMPA-PAM enhances the agonist response without direct agonist effects. AMPA-PAMs act either by attenuating desensitization, a process by which the receptor ion channel closes although glutamate remains tightly bound, and/or by slowing receptors deactivation *i*.*e*. slowing the rate at which the ion channel closes after the removal of glutamate [15, 16, 19, 20]. It is also described that AMPA-PAMs are highly sensitive to the flip or flop splice variant of AMPA receptors subunit proteins [21–24]. Overall, AMPA-PAMs stabilize the AMPA receptor in its active conformation following glutamate binding and enhance synaptic currents, thereby promoting synaptic transmission and plasticity [15, 25, 26].

Accordingly, AMPA-PAMs have been reported to facilitate induction and maintenance of long-term potentiation, a form of synaptic plasticity that is believed to underlie memory formation [3, 27, 28]. They have been reported to facilitate episodic and spatial working memories in a number of behavioural studies in rodents, monkeys and humans [15, 16, 20, 26, 29, 30]. In rodents, the novel object recognition test [31, 32] and maze-type tasks *e*.*g*. the T-maze and its spontaneous alternation behaviour assessment [33, 34] were developed to assess respectively episodic-like and working memories, both forms of memory known to be early impaired in Alzheimer’s disease patients [35]. Additionally, some AMPA-PAMs displayed neuroprotective effects shown to be linked to a release of the brain-derived neurotrophic factor (BDNF) partly involved in synaptic plasticity [29, 36–39]. Thereby, among the several classes of AMPA-PAMs described [40, 41], some molecules were developed for the treatment of schizophrenia, depression or cognitive disorders associated with Alzheimer’s disease.

Herein, we report the *in vitro* characterization of the AMPA-PAM S 47445 (8-cyclopropyl-3-[2-(3-fluorophenyl)ethyl]-7,8-dihydro-3H-[1,3]oxazino[6,5-g][1,2,3]benzotriazine-4,9-dione). The mechanism of action and selectivity of S 47445 towards some AMPA receptors subtypes were first tested on receptors expressed either in *Xenopus* laevis oocytes or in HEK-293 cells. The identification of the allosteric binding site of S 47445 was then assessed using an AMPA-kainate chimera approach. Putative neurotoxic effects of S 47445 were further evaluated using rat cortical cell cultures. As S 47445 positively modulated mature form of BDNF in dorsal hippocampus and in prefrontal cortex in aged rats [42], the involvement of this neurotrophin was also assessed in the neuroprotective effect observed at 10 μM. We finally report *in vivo* results obtained after acute or sub-chronic administrations (3 days) of S 47445 in two rodent cognitive-based models. Some CNS safety results are further provided.

## Materials and methods

### *In vitro* studies

#### Reagents

S 47445, micronized form was synthetized by Servier (WO/2008/085506), France. S 18986, LY404187 and LY451395 were provided by Servier, Suresnes, France and CX614, CX929 and CX516 by Cortex Pharmaceuticals, Irwine, USA. Cyclothiazide was obtained from Tocris Bioscience, Lille, France. MK801 was obtained from Sigma-Aldrich, Lyon, France. BDNF IgG antibodies came from Biosensis (Montluçon, France). All reagents for *in vitro* studies were obtained from Sigma-Aldrich, Lyon, France or Thermofisher Scientific, Ecubens, Switzerland or Villebon sur Yvette, France. The selective non-competitive AMPA antagonist GYKI52466 (4-(8-Methyl-9H-1,3-dioxolo[4,5-h][2,3]benzodiazepin-5-yl)-benzenamine dihydrochloride) was obtained from Tocris Bioscience, United Kingdom. Stock solutions of compounds tested *in vitro* were made in dimethylsulfoxide (DMSO) and diluted on the day of experiment in OR2 or cell culture media. DMSO concentration was inferior to 1%, which is known to have no significant effects in the experimental models tested.

#### Competition binding experiments

According to standard validated protocols by CEREP, Celle d’Evescault, France using competition binding experiments, affinities of S 47445 were evaluated in duplicate at two concentrations (0.1 and 10 μM) at AMPA, kainate and NMDA glutamatergic receptors on rat cerebral cortex membranes, using [^3^H] AMPA, [^3^H] kainate and [^3^H] CGP 39653 as radioligands, respectively. Binding of S 47445 on a panel of hundred receptors/channels/enzymes and transporters was also performed at 0.1 and 10 μM by CEREP, Celle d’Evescault, France. Experiments were performed in duplicate using competition assays on either human recombinant CHO or HEK293 cells or on rat cerebral cortex membranes. They were followed-up for IC_50_/Ki determination when compounds displayed more than 50% inhibition of control value at 10 μM. IC_50_ corresponds to the concentration of compound responsible for 50% of inhibition, Ki to the inhibition constant. Reference standards were run in parallel to ensure the validity of the measurements and results.

#### Electrophysiological recordings in *Xenopus laevis* oocytes

All animal experimentations were performed in accordance with local ethical committees following the principles of laboratory animal care with the European Communities Council Directive European Communities Council Directive 2010/63/UE or following the animal rights rule from the Geneva canton. All efforts were made to minimize animal suffering and to reduce the number of animals used.

Following anesthesia in 0.15% tricaïne methanesulfonate (MS222), a small incision was done in the lower part of the abdomen of Xenopus laevis and a cluster of oocytes was removed. Oocytes were placed at 4°C in a sterile Barth solution containing in mM NaCl 88, KCl 1, NaHCO3 2.4, HEPES 10, MgSO4.7H2O 0.82, Ca(NO3)2.4H2O 0.33, CaCl2.6H2O 0.41, at pH 7.4, and supplemented with 20 μg/ml of kanamycin, 100 unit/ml penicillin and 100 μg/ml streptomycin. On the second day after dissociation, oocytes were isolated by mechanical and enzyme dissociation using type I collagenase at 0.2% in a medium deprived of calcium with gentle mixing for a couple of hours. Oocytes were then manually selected under a binocular and injected with 50.6 nl per oocyte of rat cortex or human hippocampus poly(A+) mRNA (1 μg/μl). For experiments conducted on human AMPA subunits, oocytes were placed in a 96 microtiter plate with conical bottom and injected with 10 nl solution containing the cDNA encoding for the desired subunits at a concentration of 0.1 μg/μl using the Roboinjected (Multichannel Systems, Reutlingen, Germany). For experiments conducted on AMPA/Kainate chimera, oocytes were injected using the Roboinjected with 20 nl solution of mRNA at 0.2 μg/μl. Injected oocytes were incubated at 16–18°C for a minimum of 3 days in Barth’s solution before electrophysiological testing. They were then stored at 4°C until use (typically 1–2 weeks).

Rat cerebral cortex poly(A+) mRNA were prepared from male Wistar rats (80–100 g) by the guanidium thiocyanate/phenol/chloroform single-step method and isolated using oligo(dT)-cellulose chromatography [43]. Human hippocampal mRNA poly(A+) mRNA were provided by Clontech, Saint-Germain en Laye, France. cDNAs encoding human GluA and GluK2 subunits were provided by Origene, Herford, Germany. cDNAs encoding human GluA2 R editing form and GluK2 Q editing form were used. The ratio of GluA1 or GluA4 to GluA2 was 1:1. Chimerae between GluA1flop (GluA1o) AMPA and GluK2 kainate receptors were designed following the scheme of publications [44, 45]. Exchanges between GluA1flop and GluK2 subunits were performed on NTD and S1 segments. GluA1(K2NTD) chimera corresponded to K2(1:400)GluA1(401:929), domain of junction indicated in parentheses depicting the first and last aminoacid of each wild type subunits, and GluA1(K2S1) chimera to GluA1(1:377)K2(378:559)GluA1(560:910). Plasmids were linearized and mRNA was synthesized using mMESSAGE mMACHINE^®^ T7 Transcription Kit (ThermoFisher Scientific, Ecubens, Switzerland) following the instruction from the manufacturer.

Electrophysiological recordings were performed at 18–20°C by using two electrodes voltage clamp recordings either in a plexiglas recording chamber or by using the HiClamp automated system (Multichannel systems, Reutlingen, Germany). Oocytes were superfused with OR2 medium containing in mM: NaCl 88.5, KCl 2.5, HEPES 5, MgCl2 1, CaCl2 1.8 and Na2HPO4 1, pH 7.4. For NMDA recordings, MgCl2 was omitted from the medium. Cells were held at –60 or –80 mV. Compounds were tested by bath application. For recordings on oocytes injected with rat or human poly(A+) mRNA, multiple applications of either 10 μM AMPA, 1 mM kainate or 300 μM NMDA/30 μM glycine were first performed for 30 s with 5 min interval with a flow rate of 3 ml/min until stabilization of the evoked currents. Compounds were then tested on the same oocyte for 45 s before, 30 s during and 30 s after the application of the agonists in a concentration-dependent manner at 5 min interval. For recordings on oocytes expressing subunits of human AMPA receptors or AMPA/Kainate chimerae, application of either 300 μM or 1 mM glutamate was first performed for 20 s. S 47445 was then bath-applied on the same oocyte for 45 s before and 20 s during the application of glutamate in a concentration-dependent manner with 2.5 min interval. S 47445 was applied from 0.1 to 100 or 300 μM, Cyclothiazide from 0.3 to 300 μM, S 18986 from 3 to 1 000 μM, CX614 from 0.1 to 300 μM, CX929 from 1 to 100 μM, CX516 from 30 to 3 000 μM, LY404187 from 0.1 to 100 μm and LY451395 from 0.01 to 30 μM.

For experiments conducted on *Xenopus laevis* oocytes expressing the GluA1flop/GluA2flip AMPA receptors subtypes (i.e. GluA1o/GluA2i) and using low concentrations of S 47445 (0.1 and 0.3 μM), the glutamate-evoked currents were studied with repetitive pulses of 10 μM glutamate applied for 20 s, 1/min and S 47445 was applied after a stabilization period of 5 min. To test the inhibition, the selective AMPA receptor antagonist GYKI52446 was used from 10 to 1000 μM on the potentiation caused by S 47445 (1 μM).

Amplitudes of evoked currents were evaluated at the peak of the inward currents. The amplitude of agonist-evoked currents in the presence of AMPA-PAMs was normalized to unity *versus* the initial control response to the agonist alone evoked on the same oocyte (taken as unity). Data were filtered at 10 Hz, captured at 100 Hz and analyzed either using pclamp9 data acquisition and analysis software (Molecular Devices, Sunnyvale,USA) or HiQscreen proprietary data acquisition and analysis software running under Matlab (Mathworks Inc., Natick, USA). For GluK2 receptors data were filtered at 100 Hz and captured at 300 Hz.

The concentration of AMPA-PAMs causing 50% of the maximal effect (EC_50_) was estimated by non-linear regression based on logistic 4-parameters model where

$$\%\text{ current}=\text{First experimental value }+\frac{\left(\text{Last experimental value }–\text{ First experimental value}\right)}{1+\;10\;\left(\text{LogEC50-LogConc}\right)\text{x nH}}$$

with EC_50_ corresponding to the concentration at semi-amplitude of effect (Last_Experimental data – First_Experimental data), nH the Hill number or the slope at the inflexion point and Conc the drug concentration. EC_50_ were determined either using Matlab (Mathworks inc., Natick, USA) or SAS v9.2 (SAS Institute inc., Cary, USA) softwares. To have a comparative value between the tested drugs when EC_50_ could not be determined (absence of plateau at high concentrations), the EC_2X_ was determined. EC_2X_ corresponds to the concentrations of the drug responsible for a two-fold increase in the amplitude of the current.

Comparisons of EC_50_, EC_2X_ and maximal potentiation (Emax) were performed either using unpaired T-test or a one-way analysis of variance (ANOVA) followed by Tukey’s test under EASYSTAT software developed and validated by the Servier’s Biostatistical department, interfacing SAS v9.2 software (SAS Institute, Cary, NC) (statistical comparison performed on LogEC_50_).

#### Patch clamp recordings in HEK-293 cells

Patch clamp recordings were conducted in transiently transfected HEK-293 cells. HEK-293 cells were grown in culture medium containing DMEM GlutaMAX^™^ Supplement, pyruvate + 10% fetal bovine serum albumin + 1% Antibiotic Antimycotic Solution and transient transfection with plasmids containing GluR1o, GluR2i and GFP was conducted using lipofectamine 3000 (Thermofisher Scientific, Ecubens, Switzerland) according to the manufacturer information. Two days after transfection, cells were dissociated using Versene 1:5000 and plated on 35 mm dishes for subsequent patch clamp recordings. As HEK293 cells are electrically coupled, dissociation was indispensable to obtain enough isolated cells for patch clamp recording.

Cells expressing the GFP fluorescence were observed on an inverted microscope (Axiovert 135TV Zeiss, Oberkochen, Germany) and were probed for expression of GluR1o/GluR2i. Whole cell patch clamp recording was done at holding potential of –80 mV in a medium containing 150 mM NaCl, 4 mM KCl, 8 mM HEPES, 1.8 mM CaCl_2_, 1 mM MgCl_2_ and 20 mg/ml Bovine serum albumin adjusted to pH 7.4 with NaOH at room temperature using borosilicate glass pipettes filled with a medium containing 135 mM CsCl, 1 mM MgCl2 and 10 mM HEPES, pH adjusted to 7.2 with CsOH and presenting about 3 MΩ connected to an AxonClamp 200A amplifier (Molecular Device, Sunnyvale, USA). Data were captured with an analog to digital converter (National Instrument), recordings were done at 2 KHz and off line analysis were done with a proprietary software written in Matlab (Mathworks, inc., Natick, USA). Drug application was done using a liquid filament formed at the extremity theta tube mounted on a piezo quartz (Physics Instrument, Karlsruhe, Germany) allowing a fast application as previously described [46].

#### Toxicity on rat primary cortical cell cultures

Both intrinsic S 47445-evoked toxicity and effect of S 47445 on glutamate-mediated toxicity were evaluated after 24 h incubation on primary rat cortical cells cultures by determination of lactate deshydrogenase (LDH) release in culture medium (marker of damaged cells). LDH is a stable soluble cytosolic enzyme that cannot cross the cellular membrane and is released into the culture medium following loss of membrane integrity as a results of cell damage and cytotoxic effects.

Pregnant female Wistar rats (Janvier Labs, Le Genest Saint Isle, France) of 16–17 gestation were sacrified by cervical dislocation and the fetuses were removed from the uterus. Their brains were placed in ice-cold medium of Leibovitz’s L-15 medium, Gibco, Fisher bioblock, France. Cortex was dissected and meninges were carefully removed. The cortical neurons were dissociated by trypsinization for 30 min at 37°C (trypsin-EDTA, Gibco) in presence of 0.1 mg/ml DNAse I (Roche, France). The reaction was stopped by addition of Dulbecco’s modified Eagle medium (DMEM), Gibco with 10% of fetal bovine serum, Gibco. The suspension was triturated with a 10-ml pipette and using a needle syringe 21G and centrifuged at 350 x g for 10 min at room temperature. The pellet of dissociated cells was resuspended in a medium consisting of Neurobasal (Gibco) supplemented with 2% B27 supplement (Gibco), 0.5mM L-Glutamine (Gibco) and an antibiotic-antimicotic mixture. Viable cells were counted in a Neubauer cytometer using the trypan blue exclusion test (Sigma).

Dissociated rat cortical cells were seeded on the basis of 35 000 cells per well in 96-well culture plates and were maintained in a humidified incubator at 37°C in 5% CO2-95% air atmosphere. Four independent cultures with replicates (n = 4) were performed. The effect of S 47445 against glutamate-induced neuronal damage was studied following a concentration-response curve exposition of glutamate (0.03 to 100 μM). On day 12 (24h before glutamate application), the cultures were placed for 24h in fresh medium containing either vehicle or S 47445 (0.1-3-10 μM) or MK801 (1 μM, Sigma-Aldrich), a NMDA channel blocker used as positive reference control. On day 13, rat primary cortical neurons were exposed to a concentration-response of glutamate (0.03, 1, 3, 10, 20, 50, 75 and 100 μM) for 10 min. Cultures were then washed with DMEM and placed in fresh medium supplemented with either vehicle or test compound for a further 24h. Then, S 47445 (10 μM) was also tested in combination with a neutralizing IgG BDNF antibody (10 μg/ml, Biosensis).

Neuronal damage were assessed by measuring LDH release in the media 24h after glutamate exposure using the CytoTox 96^®^ non-radioactive kit (Progema, Charbonières, France). CytoTox 96^®^ is a colorimetric enzymatic assay system that measures the conversion of a tetrazolium salt into a red formazan product by LDH. Visible wavelength adsorbance data will be collected using a 96 well plate reader at 450 nm (Labsystems Multiskan Bichromatic, Cergy Pontoise, France). The percentage of LDH activity was determined using control or untreated glutamate-intoxication condition as reference and was normalized to response of the vehicle group taken as 100%.

### *In vivo* studies

#### Animals

All animal experimentations were performed in accordance with local ethical committees following the principles of laboratory animal care with the European Communities Council Directive 2010/63/UE. All efforts were made to minimize animal suffering and to reduce the number of animals used.

Novel object recognition and T-maze studies were conducted respectively in male OFA rats (150–175 g, Charles River, Saint-Germain-Nuelles, France) and male C57Bl/6 mice (5.5–6.5 month-old, Charles River, Saint-Germain-Nuelles, France). Animals were housed in collective cages with continuous access to tap water and food on a 12 h light-dark cycle, in a temperature-controlled (21 ± 1°C) and ventilated room. On completion of studies subjects were euthanized by either CO2 inhalation or intraperitoneal (i.p.) lethal pentobarbital injection. The novel object recognition test was used as the primary *in vivo* screening assay [32] to identify AMPA-PAMs. The T-maze task was performed using a modified continuous performance procedure previously described in mice [33].

#### Drugs

S 47445 was suspended either in distilled water with Tween80^®^ 2% for oral administration or saline with Tween80^®^ 2% for i.p. administration. Tween80^®^ was obtained from Sigma-Aldrich, Lyon, France. S 47445 was synthetized by Servier, Suresnes, France. Detailed data results are available in supplementary information.

#### Novel object recognition test in rats

The novel object recognition paradigm was developed by Ennaceur and Delacour, 1988 and aimed to evaluate potential procognitive effects of drugs on a form of recognition (episodic-like) memory in the rat. This test is based on a natural behaviour of rodents *i*.*e*. a greater spontaneous exploration of a novel object as compared to a familiar object. Consequently, recognition is measured by the time spent by rats in exploring two different objects, one being familiar and the other being new. With an inter-trial interval of 2 h, normal rats spend more time exploring the new object which demonstrates that they recognize the familiar one. After a retention interval of 24 h, the rats do not discriminate between the two objects as indicated by similar times spent in exploring each of them.

Animals were submitted to three phases of the test. The first phase was a 3-min habituation period with a free exploration of the empty experimental apparatus comprising an open-field made of grey Plexiglas (65 x 45 x 42 cm). During the second phase (session of familiarisation), two identical objects were placed on the floor in the same apparatus. Each rat was placed in the arena, and the time spent actively exploring the objects was recorded. Rats were removed as soon as they reached 15 sec of total exploration of the objects. After 24 h, during the third phase (session of recognition), each rat was again placed in the apparatus for 3 min in the presence of one of the familiar (Fam) objects and a new (New) object, and the time spent exploring both objects was again recorded. Rats were orally treated with either S 47445 at 0.3, 1 or 3 mg/kg, or vehicle daily for 3 days *i*.*e*. 60 min before the habituation, familiarisation and recognition phases. The difference in duration of exploration of the two objects (New-Fam) and the sum of duration of exploration on session of recognition were determined. Statistical analysis was performed on the discrimination index calculated as (New-Fam)/(Fam+New) using a one-way ANOVA using SAS v9.2 software (SAS Institute, Cary, NC). If statistically significant (p≤0.05), ANOVA was followed by Dunnett’s test to compare S 47445-treated groups *versus* vehicle controls. Analyses were performed using SAS v9.2 software (SAS Institute, Cary, NC).

#### Spontaneous alternation in a T-maze in mice

Spontaneous alternation was developed regarding the intrinsic motivation of rodents to explore new environments. In practice, mice are given the choice of exploring each of both arms of a T-shaped maze. Natural tendency of a rodent is to visit successively one arm after the other (alternation phenomenon) in particular if the delay between each visit is short. A longer delay between each visit (180 sec) induces a lower alternation score resulting from an increased level of interference between trials in vehicle-treated animals [33]. Procognitive compounds [47] should reduce interference between successive trials as compared to vehicle, thereby increasing alternation level. This effect reflects a performance of spatial working memory.

Behavioral testing was conducted in a T-shaped maze made of grey Plexiglas using mice. The central alley (35 x 15 x 10 cm) was connected to a start box and to two “arrival” areas (same dimensions: 15 x 10 cm). Photoelectric cells detected the position of the mouse on the maze. Opening and closing of three sliding doors (one at the exit of start box, two at the entry into goal-arms) were piloted by software (Imetronic, Pessac, France).

Briefly, mice were firstly given 10-min daily sessions (3 days) for free exploration in the apparatus. Sliding doors were lowered, and the mouse could enter into any arm and return onto the central alley. Mice were then submitted to 8 successive trials. For each trial, the mouse was placed in the start box for a 180 sec-delay (excepted at the 8th trial for which the delay amounted to 30 sec). At the end of this delay, the sliding door was lowered, and the mouse could move into the central alley. As soon as the animal freely entered into one of the two arms, the sliding door was raised. The mouse remained in the chosen arm for 30 sec. At the end of this period, the mouse was manually grasped by the experimenter and placed back into the start box for a new trial. The effect of a single administration of S 47445 (0.1, 0.3 and 1 mg/kg i.p.) on memory performance was investigated as compared to vehicle. Treatments were administered 30 min prior to testing.

Both alternation performance and latency of entry were assessed. An alternation was defined as a visit in a given arm followed by a visit into the other arm. The successive sequence of visits for the 7 first trials determined the level of alternation. A percentage of alternation was calculated for each mouse, and corresponded to the number of alternations performed relative to the number maximal of alternation (n = 6). For study validity, vehicle alternation rates must be within a 50 ± 9% for a 180 s inter-trial delay. The latency of entry was the time elapsed between the exit from the start box and the entry into a chosen arm (sec). The mean latency of entry from trial 2 to trial 7 was calculated for each mouse. Statistical analysis was performed on the percentage of alternation and on the mean latency of entry using a one-way ANOVA using SAS v9.2 software (SAS Institute, Cary, NC). If statistically significant (p≤0.05), ANOVA was followed by Dunnett’s test to compare S 47445-treated groups *versus* vehicle controls. Analyses were performed using SAS v9.2 software (SAS Institute, Cary, NC).

#### Spontaneous locomotor activity in mice

Locomotor activity was measured in male NMRI mice (18–20 g, Janvier Labs, Le Genest Saint Isle, France) placed in open-fields equipped with infrared sensors (Imetronic, Pessac, France). Two parameters were automatically computed: the global locomotor activity and the number of rearing by counting the number of times the animal cut photocells. Mice were placed in the apparatus 30 min after oral administration of S 47445 (10, 30 and 100 mg/kg) or vehicle. Spontaneous locomotor activity was recorded for 60 min divided in 6 x 10-min periods. The global locomotor activity was analysed using two way ANOVA (dose x time) followed by Dunnett’s post hoc test. Analyses were performed using SAS v9.2 software (SAS Institute, Cary, NC).

#### Spontaneous locomotor activity in rats

Locomotor activity was recorded in male adult Wistar rats (180–200 g, Janvier Labs, Le Genest Saint Isle, France) placed in cages in a soundproof keeping room (open-field). A remote surveillance appliance allowed to observe and to record animal behaviour from an adjacent room. The movie camera was connected to an automatic analysis system (videotrack software, Viewpoint, Lissieu, France). The distance covered (cm) was automatically computed for each rat. Rats were orally administered 1 h before the test with S 47445 (10, 30 and 100 mg/kg) or vehicle. Locomotor activity was recorded during 30 min divided in 3 x 10-min periods. The total covered distance over 30 min was analysed using one way (dose) ANOVA, followed in case of significance of the dose effect by Dunnett’s test (versus vehicle controls). The significance threshold was set at 5%. Analyses were performed using SAS v9.2 software (SAS Institute, Cary, NC).

#### Functional observation battery in mice

The effect of 47445 after high doses administration was assessed on general behaviour in mice [48]. Male NMRI mice (18–22 g, Janvier Labs, Le Genest Saint Isle, France) were used and placed individually for habituation, 15 min before administration. Room temperature was maintained at 20.9°C during experimentation. At T0 min, body temperature was monitored via a rectal probe connected to a computer (Viewpoint). Mice were then orally treated with S 47445 (30, 100 and 300 mg/kg) or vehicle. Mice were observed for changes in autonomic function (lacrimation, salivation, piloerection), awareness (hypo- or hyper-reactivity) and body temperature before and every 30 min up to 2 h after treatment administration. Delta of body temperature compared to T0 min (°C) was calculated. Groups were compared for body temperature at T0 min with a one-way ANOVA followed by Tukey’s pair-wise comparison post hoc tests. The treatment effect on delta of body temperature compared to T0 min was analysed using a two-way ANOVA followed by Dunnett’s post hoc tests. Analyses were performed using SAS v9.2 software (SAS Institute, Cary, NC).

#### Functional observation battery in rats

After acute oral administration of S 47445, rats were observed for potential neurobehavioural effects using a standard observation battery which allows the assessment of peripheral and central nervous systems activities (e.g., motor activity, behaviour, co-ordination, somatic sensory/motor reflex responses and autonomic responses such as piloerection, pupil size, lachrymation, salivation, overt cardiovascular and gastrointestinal effects). Any effects on body temperature were also assessed. Methods were adapted from those described by Irwin, 1968 for detecting behavioural effects in mice.

Male Wistar rats (Depré breeding centre, France), 6 weeks of age (160.0–190.6 g) on the day of randomisation were used. On the day of the test, animals were first scored using the Irwin standardised observation battery, and the body temperature was measured to establish baseline measurements. Groups of rats were then orally treated with S 47445 (50, 200, 500 and 1000 mg/kg) or its vehicle or clonidine, a reference item (3 mg/kg in water). The Irwin scores as well as measurement of body temperature were performed again at 1, 2, 4, 6 and 24 hours after administration of S 47445. Individual observation data were presented as a score attributed to each behavioural sign (intensity and relevance). For observations with absence or presence, the number of animals with the sign was provided per group. For other observations, the median value for each sign per time was presented per group. For body temperature, data were expressed as variations from predose values. The effects of active treatments on each behavioural sign were compared to those of vehicle using a non-parametric Mann-Whitney U test or a Fisher’s test or an analysis of variance for repeated measurements. The treatment effect on body temperature was analysed using one way ANOVA followed by Newman-Keul’s post hoc test. Analyses were processed using RS/1 software (release 6.3, Applied Materials, Santa Clara, USA).

## Results

### S 47445 is a positive allosteric modulator selective for AMPA receptors

**Fig 1:**
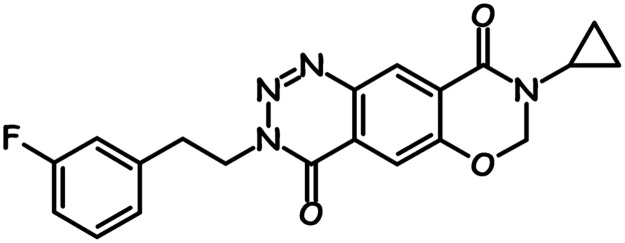
Chemical structure of S 47445. Chemical name of S 47455 is 8-cyclopropyl-3-[2-(3-fluorophenyl)ethyl]-7,8-dihydro-3H-[1,3]oxazino[6,5-g][1,2,3]benzotriazine-4,9-dione.

S 47445 is a molecule discovered through collaboration between Cortex Pharmaceuticals and Servier (Fig 1), which did not display affinity for the glutamate binding site on AMPA, kainate or NMDA receptors (Ki values greater than 10 μM, n = 2). In order to check the selectivity of S 47445 and to detect a potential for adverse effects, S 47445 was evaluated *in vitro* on about a hundred receptors, enzymes, transporters and channels. The percentage of inhibition of control value in presence of 10 μM S 47445 was lower than 50%, indicating that Ki values in all these assays were greater than 10 μM (S1 Table), thus suggesting no affinity of S 47445 for these studied receptors, enzymes, transporters and channels.

**Fig 2:**
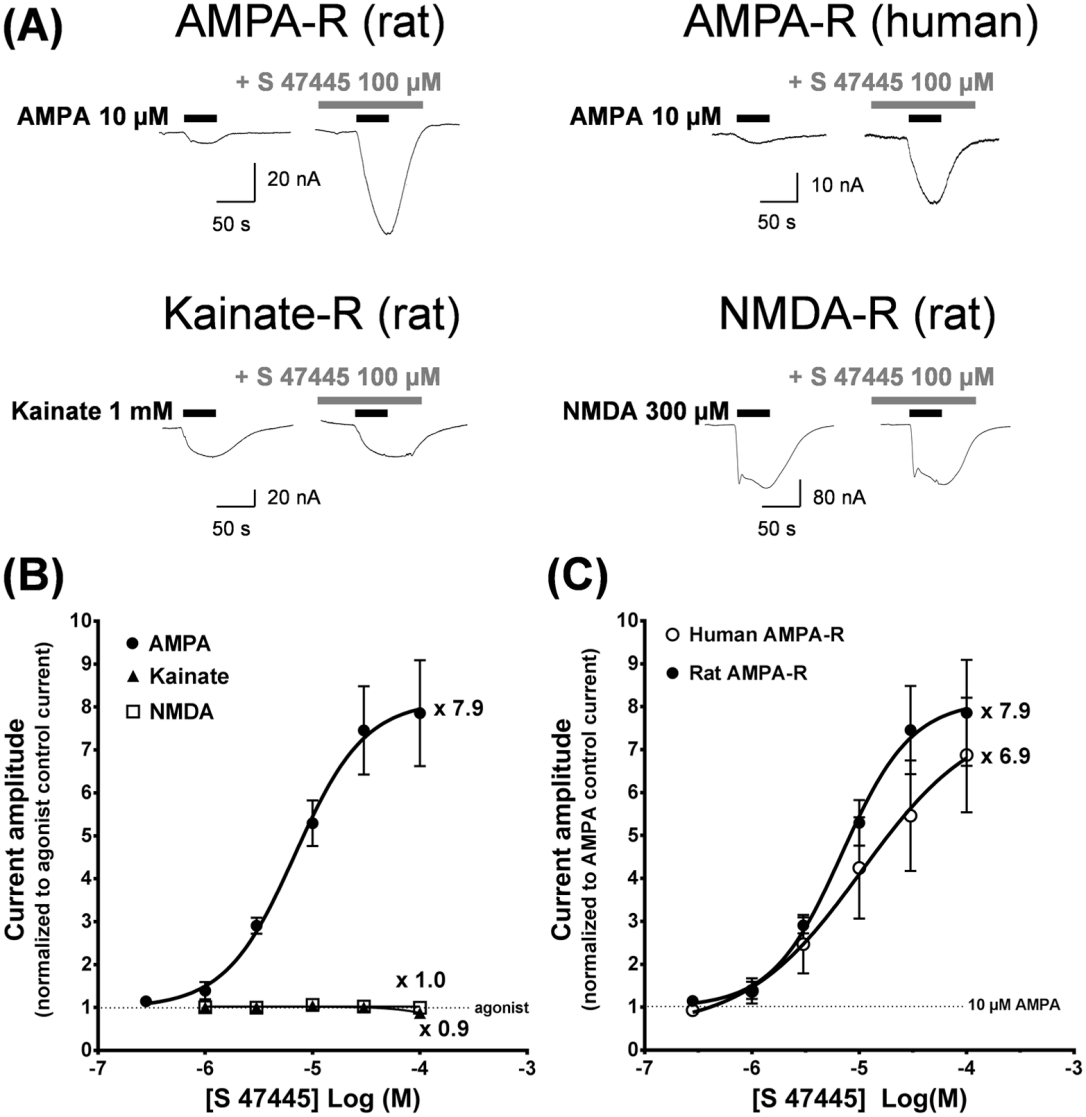
Effects of S 47445 on native rat and human AMPA, NMDA or kainate receptors. (A) Typical currents evoked by AMPA, kainate or NMDA on oocytes injected with rat cortex or human hippocampal poly(A+) mRNA in absence or presence of 100 μM S 47445 recorded in the same cell. (B) Concentration-activity curves obtained with S 47445 on AMPA-, kainate- or NMDA-evoked current on oocytes injected with rat cortex poly(A+) mRNA (n = 3, 3 and 4, respectively). (C) Comparative effects of S 47445 on AMPA-evoked current on oocytes injected with either human hippocampal or rat cortex poly(A+) mRNA (n = 5 and 3, respectively). In (A), (B) and (C), cells were held at -60 mV. The amplitude of current evoked in the presence of S 47445 was normalized to unity versus the control current evoked by the agonist alone on the same oocytes (either 10 μM AMPA, 1 mM kainate or 300 μM NMDA/30 μM glycine). Results are expressed as mean ± SEM.

S 47445 had no effect on the holding current of *Xenopus laevis* oocytes injected with rat cortex mRNA, but enhanced AMPA-evoked inward currents up to about 8 fold in a concentration-dependent manner with an EC_50_ of 6.5 μM (n = 3, see representative signals on Fig 2A and 2B, Table 1). Potentiation evoked by S 47445 is selective for the AMPA-evoked currents as shown by the absence of effect at NMDA- or kainate-evoked currents (n = 3, Fig 2A and 2B). Similar experiments were conducted on oocytes injected with human hippocampal poly(A+) mRNA and compared to rat data (Fig 2C, Table 1). S 47445 caused no detectable modification of the holding current but potentiated up to about 8 fold the amplitude of the response evoked by AMPA (n = 5). Importantly, both human and rodent AMPA receptors displayed equivalent EC_50_’s for S 47445 and similar magnitude of potentiation indicating that results obtained in rodents can be extrapolated to humans (p>0.05, unpaired T-test). Similar observations were obtained with other positive allosteric modulators that displayed similar activities in both species (Table 1). Regarding EC_50_ values (or EC_2X_ for CX929 and CX516 as EC_50_ values could not be determined), S 47445 is more potent than S 18986, CX516, CX614 and CX929, less potent than LY451395 and equivalent to cyclothiazide and LY404187. Larger maximally evoked potentiations were also observed following S 18986, LY404187, LY451345 and CX614 application compared to S 47445 (Table 1).

**Table 1:** EC_50_, EC_2X_ and maximal potentiation values obtained with S 47445 and other positive allosteric modulators at rat or human AMPA receptors on oocytes injected with poly(A+) mRNA.

	Rat AMPA receptors			Human AMPA receptors		
	EC_50_ (μM)	EC_2X_ (μM)	E_max_ (fold increase)	EC_50_ (μM)	EC_2X_ (μM)	E_max_ (fold increase)
**S 47445**	6.54 (n = 3)	1.53	7.85 ± 1.23	7.08 (n = 5)	4.77	6.87 ± 1.33
	[0.34; 126.00]	[1.32; 1.77]		[2.82; 17.80]	[0.67; 34.17]	
**Cyclothiazide**	8.83 (n = 5)	1.68	8.86 ± 0.79	9.33 (n = 3)	1.54	9.40 ± 2.61
	[4.34; 17.99]	[1.09; 2.59]		[1.40; 62.27]	[1.22; 1.94]	
**S 18986**	125.31 (n = 5)	23.77	15.37 ± 2.21	96.6 1 (n = 4)	16.88	14.51 ± 1.53
	[85.10; 184.53]	[16.40; 34.44]		[83.06; 112.36]	[7.70; 37.04]	
**LY404187**	3.01 (n = 5)	0.32	16.40 ± 2.22	4.64 (n = 3)	0.37	29.67 ± 2.67
	[2.21; 4.08]	[0.25; 0.41]		[3.80; 5.85]	[0.20; 0.70]	
**LY451395**	0.37 (n = 3)	0.04	14.03± 0.70	na	na	na
	[0.05; 2.89]	[0.02; 0.07]				
**CX614**	17.30 (n = 5)	1.03	30.32 ± 5.67	11.39 (n = 3)	0.88	23.43 ± 2.32
	[8.01; 37.36]	[0.81; 1.32]		[5.07; 25.60]	[0.38; 2.06]	
**CX929**	nd (n = 4)	16.79	5.37 ± 0.64	nd (n = 5)	22.55	4.93 ± 1.41
		[7.73; 36.47]			[6.94; 72.91]	
**CX516**	nd (n = 6)	nd (n = 6)	1.72 ± 0.1	nd (n = 2)	1 230.04	3.13

EC_50_ and EC_2X_ values are expressed as geometric mean and confidence intervals and maximal potentiation (Emax) as arythmetric mean ± SEM.

nd: not determined (no EC_50_ could be determined, since no plateau was obtained at the highest tested concentrations); na: not available.

**Fig 3:**
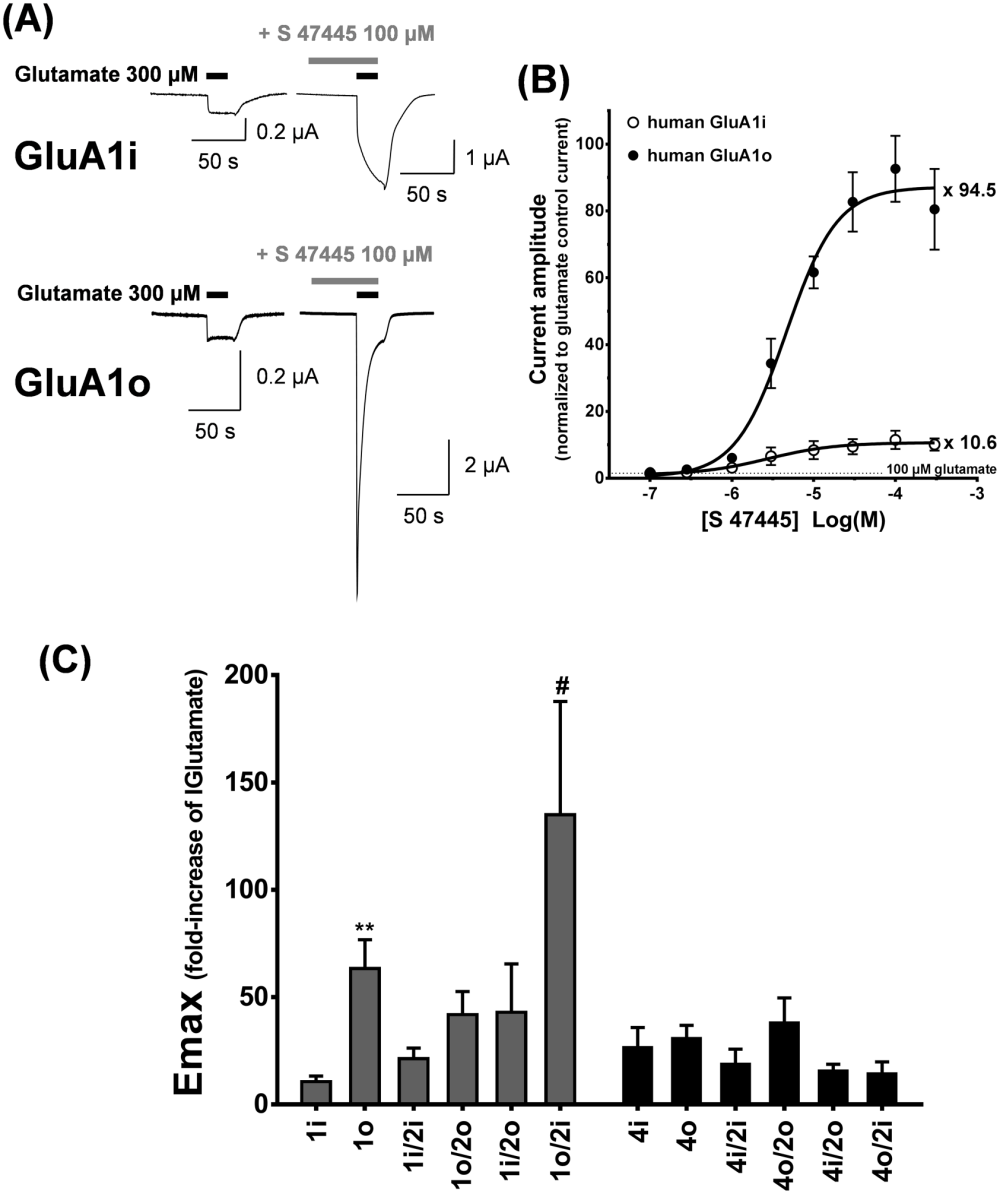
GluA1, GluA2 and GluA4 subunits and splice variants selectivity of S 47445 at human AMPA receptors. (A) Typical examples of glutamate-evoked currents in absence and presence of 100 μM S 47445 obtained on the same oocyte expressing either GluA1 flip (GluA1i, n = 5) or flop (GluA1o, n = 3) variants. (B) Concentration response curves for S 47445 obtained in oocytes injected with GluA1i and GluA1o receptors. (C) Maximal fold potentiation (E_max_) induced by S 47445 on homomeric GluA1 flip (GluA1i), GluA1 flop (GluA1o), GluA4 flip (GluA4i), GluA4 flop (GluA4o) and heteromeric GluA1/GluA2 or GluA4/GluA2 receptors. E_max_ is given as the amplitude of current evoked in the presence of S 47445 normalized to unity versus the control current evoked by glutamate alone on the same oocytes. n is indicated in brackets. ** p≤0.01 GluA1o versus GluA1i, unpaired T-test. # p≤0.05 GluA1 flop/ GluA2 flip (GluA1o/2i) versus GluA1 flip/ GluA2 flip (GluA1i/2i), Tukey’s test following significant one-way ANOVA performed on GluA1/GluA2 heterodimers. In (A), (B) and (C), cells were held at -80 mV. Values are mean ± SEM.

Selectivity of S 47445 for different AMPA receptors subtypes was evaluated using recombinant human AMPA receptors using GluA1, GluA2 and GluA4 flip and flop variants expressed in *Xenopus laevis* oocytes. As some difficulties occurred to evoke strong and reproducible glutamate responses on homomeric GluA2 receptors, we decided to assess GluA2 as heteromeric complexes with GluA1 and GluA4 subunits. No functional receptors could be obtained using human GluA3 or GluA3/GluA2 AMPA receptors subtypes in our conditions. In oocytes expressing homomeric GluA1 and GluA4 flip and flop splice variants (Fig 3A and 3B), S 47445 potentiated in a concentration-dependent manner currents evoked by a saturated concentration of glutamate (300 μM), EC_50_ values shown in Table 2. EC_50_ values did not differ among most GluA recombinants (varying between 2.5–5.4 μM), but a significant difference was observed for receptor containing the GluA4 flop subtype which presented a lower EC_50_ value (0.7 μM) compared to the GluA4 flip subtype (Table 2, p≤0.001, unpaired T-test). Moreover, the amplitude of potentiation evoked by S 47445 (100 μM) was consistently larger for flop variants (about 9-fold) compared to flip variants at GluA1 AMPA receptors (Fig 3A and 3C). Altogether these data illustrate that S 47445 acts as a powerful allosteric modulator at all human AMPA subunits tested in the present study but with a greater amount of potentiation on GluA1 flop variants.

**Table 2:** EC_50_ values obtained on different GluA1, GluA2 and GluA4 subunits and flip (i) and flop (o) splice variants at human AMPA receptors.

	EC_50_ (μM)		EC_50_ (μM)
**GluA1i**	5.4 (n = 5)	**GluA4i**	3.0 (n = 8)
	[1.2; 24.7]		[1.8; 5.2]
**GluA1o**	2.5 (n = 7)	**GluA4o**	0.7*** (n = 12)
	[1.0; 6.0]		[0.5; 1.1]
**GluA1i/2i**	2.6 (n = 7)	**GluA4i/2i**	5.4 (n = 4)
	[2.0; 3.5]		[2.8; 10.4]
**GluA1o/2o**	3.9 (n = 6)	**GluA4o/2o**	2.9^#^ (n = 5)
	[2.0; 7.8]		[2.3; 3.6]
**GluA1i/2o**	4.3 (n = 3)	**GluA4i/2o**	3.3 (n = 4)
	[0.9; 20.7]		[1.9; 5.6]
**GluA1o/2i**	5.4 (n = 5)	**GluA4o/2i**	3.8 (n = 4)
	[2.8; 10.2]		[2.8; 5.1]

Values are expressed as geometric mean and confident intervals.

*** p≤0.001 GluA4 flop (GluA4o) *versus* GluA4 flip (GluA4i), unpaired T-test performed on log EC_50_.

^#^p≤0.05 GluA4 flop/ GluA2 flop (GluA4o/2o) *versus* GluA4 flip/ GluA2 flip (GluA4i/2i), Tukey’s test following significant one-way ANOVA performed on GluA4/GluA2 heterodimers and on log EC_50_.

**Fig 4:**
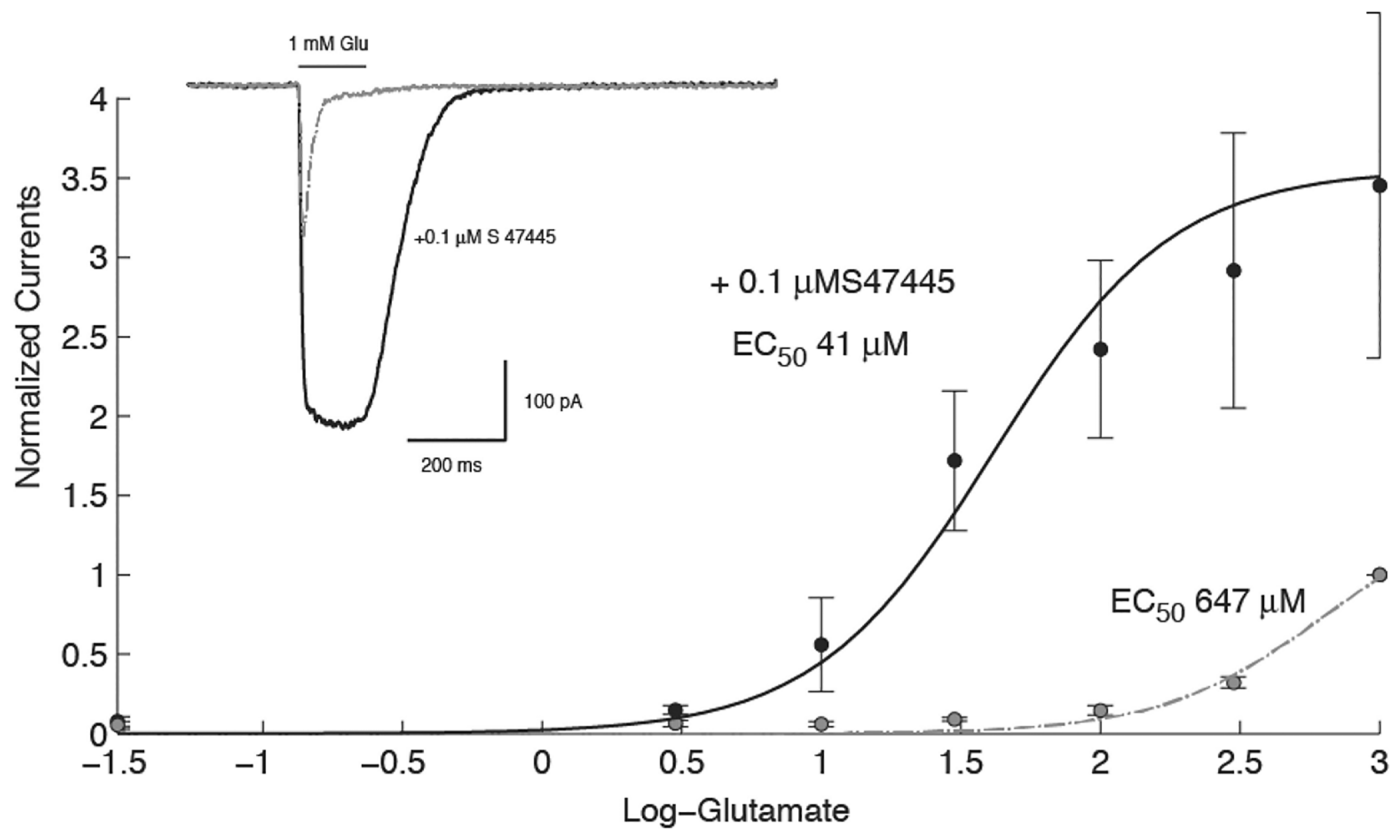
Potentiation of the glutamate-evoked current in AMPA receptors expressed in HEK-293 cells. Typical current recorded in the whole cell configuration in response to a brief pulse of glutamate in control (gray line) and during exposure to S 47445 (black line). These data illustrate that exposure to a low concentration of S 47445 causes already a significant modification of the decay time of the response. Cell was held at -80 mV. Plot of the concentration activation curve recorded in control and in presence of 0.1 μM S 47445 illustrates that this compound causes a left shift of the curve and increase of the maximal amplitude (n = 4).

To further evaluate the effects of S 47445, patch-clamp recordings were conducted on AMPA receptors transiently expressed in HEK-293 cells. Based on the distribution of AMPA receptors in the brain and especially in the hippocampus [9, 21, 49], the complex GluA1flop/GluA2flip (GluA1o/2i) was retained for these experiments. Transient transfection of GluA1flop/GluA2flip yielded functional AMPA receptors displaying fast inward currents in response to a brief glutamate test pulse. As shown in Fig 4 exposure to a low concentration of S 47445 (0.1 μM) markedly enhanced the amplitude of the glutamate-evoked current and prolonged the response. Determination of the concentration activation curve to glutamate conducted in absence and presence of 0.1 μM of S 47445 illustrates, as expected for an allosteric modulator, a left shift of the concentration activation curve from 647 ± 21.9 to 41 ± 10.9 μM (n = 4, Fig 4) accompanied by an increase in the amplitude of the response from 94.7 ± 32.8 to 247 ± 70 pA. This corresponds to the consensus hallmark of a positive allosteric modulator on a ligand gated ion channel since exposure to such compound should shift the concentration activation curve to the left, increase the steepness of the curve and the amplitude of the response [50].

Since procognitive effects was observed from the concentration of 0.1μM of S 47445, expression of GluA1flop/GluA2flip AMPA receptors subtypes in *Xenopus laevis* oocytes was further used to explore the time course of S 47445 effects around this concentration.

**Fig 5:**
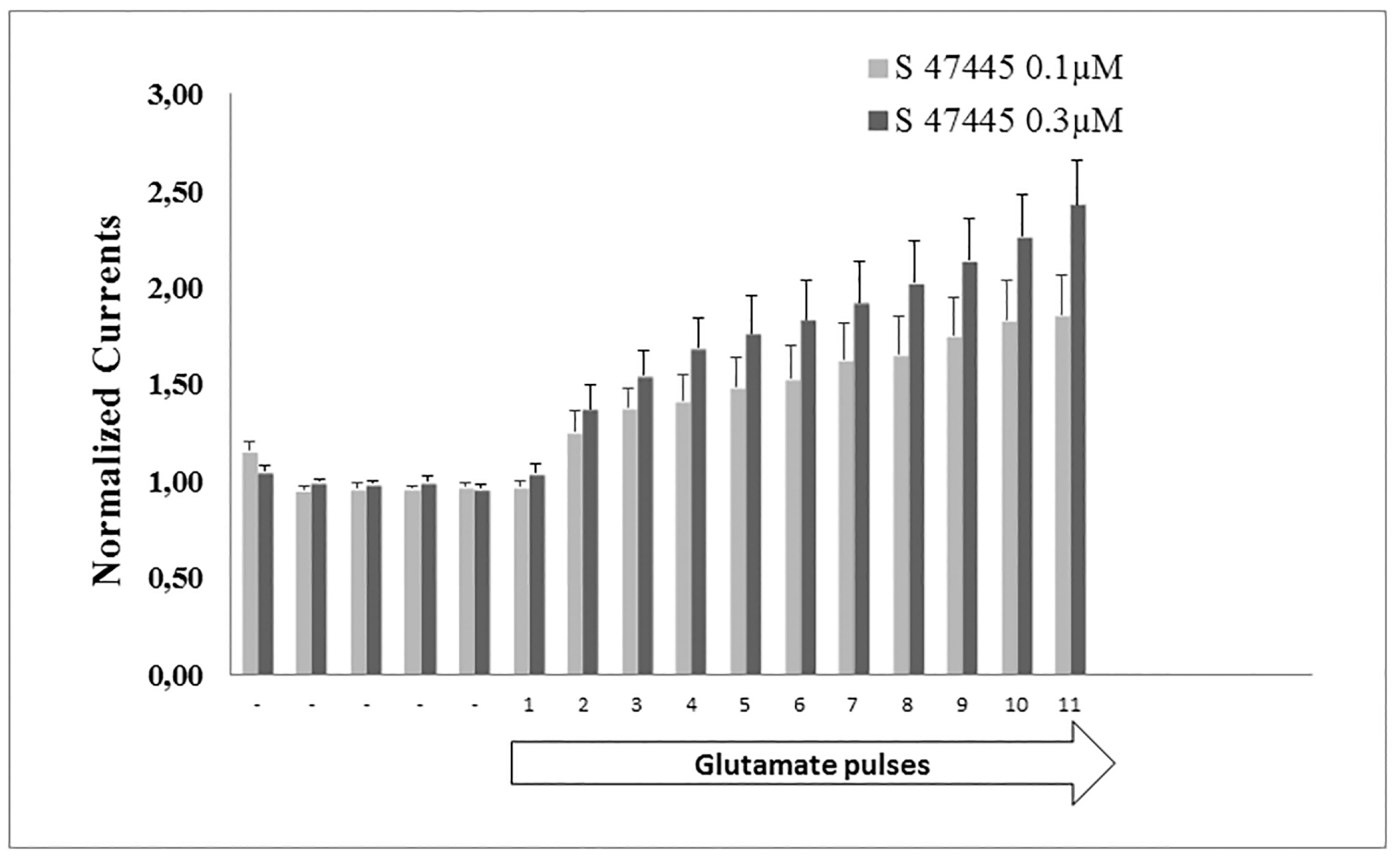
Potentiation of the glutamate-evoked current in GluA1flop/GluA2flip AMPA receptors expressed in Xenopus oocytes. Normalised currents in response to repetitive pulses of 10 μM glutamate applied for 20 s, 1/min and during exposure to low concentrations of S 47445 (0.1 and 0.3 μM). Currents were normalized to the mean of the first 5 recordings in vehicle conditions illustrated by the symbol (-). These data illustrate that exposure to low concentrations of S 47445 causes a rapid increase of potency of the AMPA receptors currents with no development of desensitization following several pulses of glutamate.

Exposure to low concentrations of S 47445 far below the EC_50_ (0.1 and 0.3 μM) applied in the presence of repetitive glutamate pulses induced a progressive potentiation of the glutamate-evoked currents from the second pulse of glutamate (Fig 5). This fast-acting effect of S 47445 on AMPA receptors currents was not accompanied by the development of desensitization following several pulses of glutamate.

### Modulatory effect of S 47445 on glutamatergic AMPA receptors and determination of the allosteric binding site of S 47445 at AMPA receptors

**Fig 6:**
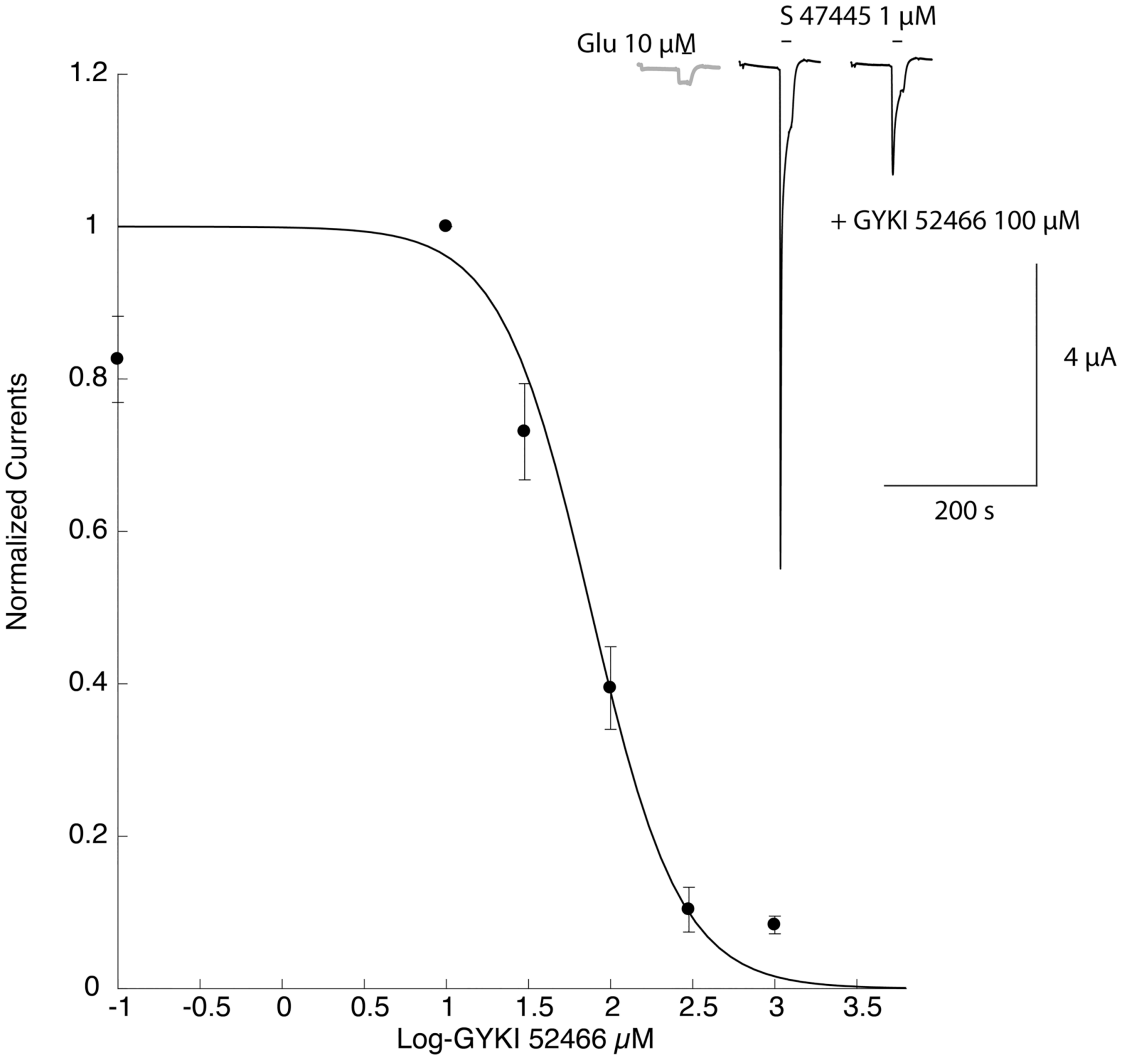
Mean concentration-response to GYKI52466 in presence of 1 μM S 47445. Typical currents in a cell expressing are shown in the upper panel and concentration-response curve in the lower panel. These data show that of the potentiation effect of S 47445 observed at the concentration of 1 μM on the amplitude of the glutamate-evoked (10 μM) was reduced by the selective AMPA receptor antagonist GYKI52466 applied from the 10 μM concentration.

In order to confirm the mechanism of action of S 47445 on the AMPA receptors, we assessed effect of S 47445 in presence of the selective AMPA receptor antagonist GYKI52466 on *Xenopus laevis* oocytes expressing the GluA1flop/GluA2flip AMPA receptors subtypes. The potentiation effect of S 47445 observed at the concentration of 1 μM on the amplitude of the glutamate-evoked (10 μM) was concentration-dependently reversed by GYKI52466 applied from 10 to 1000 μM confirming the positive modulatory effect of S 47445 on AMPA receptors (Fig 6).

**Fig 7:**
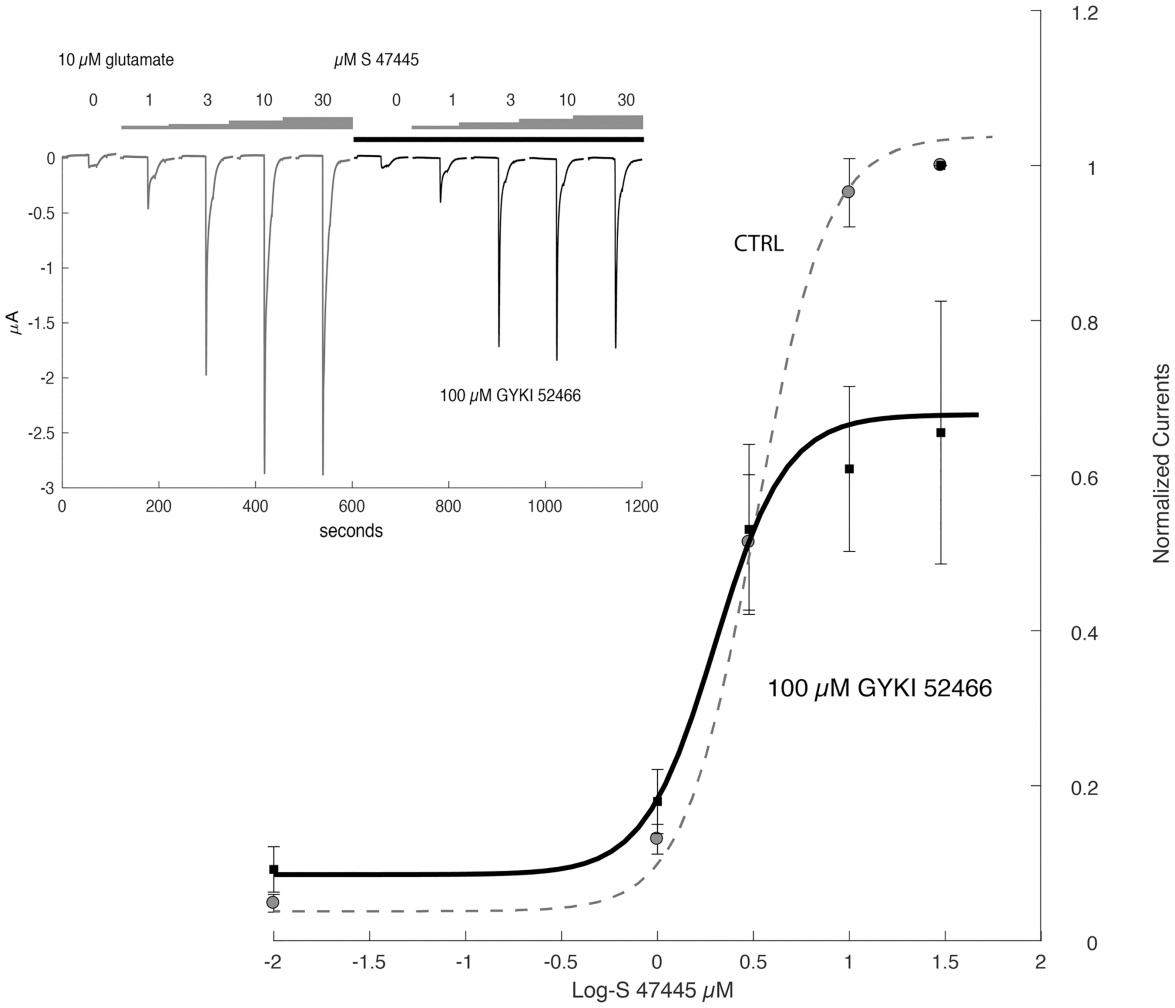
Effects of GYKI52466 attenuates the potentiation caused by S 47445 but not the sensitivity. Determination of the potentiation caused by a series of S 47445 on the response evoked by 10 μM glutamate conducted in absence or presence of GYKI52466. Typical currents evoked by a 10 μM glutamate test pulse recorded in the same cell are illustrated in the inset. Currents were normalized to unity for the maximal value recorded in presence of 30 μM glutamate alone on the same oocytes (n = 3). Note that exposure to GYKI52466 caused a significant reduction of the magnitude of the potentiation but does not alter the sensitivity to S 47445.

Furthermore, determination of the potentiation caused by a series of S 47445 concentrations on the glutamate-evoked current, in absence or presence of 100 μM GYKI52466 revealed that exposure to this compound caused a reduction of the amplitude of the potentiation but no significant shift in sensitivity (Fig 7), and comparable EC_50_ values (3.23 ± 0.5 and 1.99 ± 0.3 μM in absence and presence of 100μM of GYKI52466, respectively). The absence of a detectable shift in sensitivity further indicated that GYKI52466 and S 47445 are interacting at independent binding sites.

**Fig 8:**
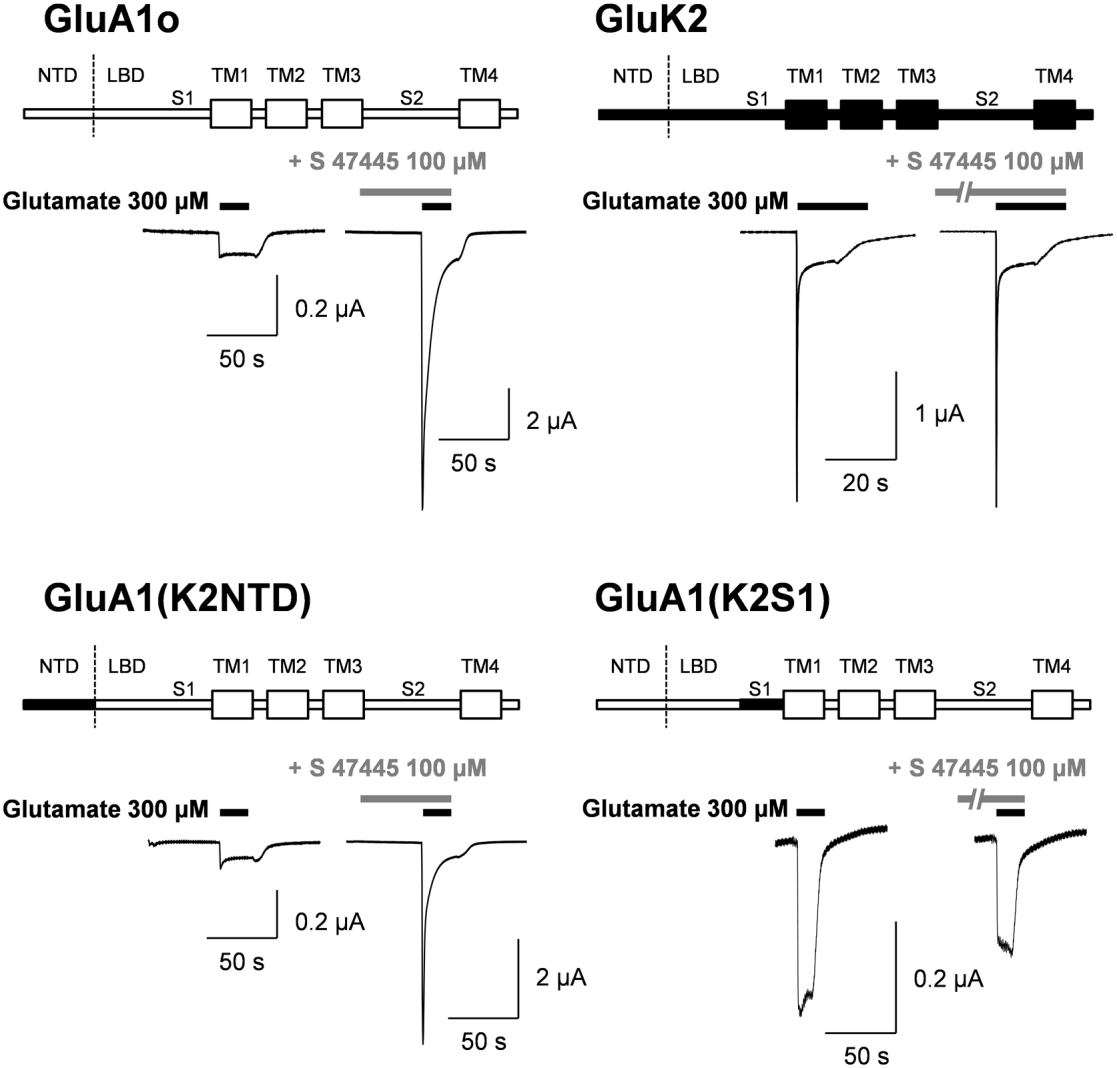
Effect of S 47445 at chimeric AMPA/kainate receptors. Typical examples of glutamate-evoked currents in absence and presence of 100 μM S 47445 obtained from the same oocyte expressing either GluA1flop (GluA1o) AMPA R receptors, GluK2 kainate Q editing form receptors or chimeric of AMPA/kainate receptors, GluA1(K2S1) or GluA1(K2NTD). Cells were held at -80 mV.

S 47445 had been tested in crystallographic experiments using human GluA2 with the corresponding amino-acids T415-S796 (Δ528–652) produced in Artic bacteria. However due to poor solubility, S 47445 did not successfully co-crystallize with GluA2 and did not allow us to determine the structures of the complexes. Therefore, taking advantage of the selectivity of S 47445 for the AMPA over the kainate receptors, the approach using chimeric proteins encompassing segments from the AMPA and the kainate receptors was investigated in order to dissect the determinant portions of the receptors contributing to the allosteric modulation of S 47445. Based on published data it is well established that fusion proteins can be made between these two receptor subtypes resulting in functional homomeric receptors containing elements from one type exchanged with their counter part from another glutamate receptor subtype [44, 45]. In fact, results obtained from crystallography studies suggest that AMPA-PAMs bind at the interface between two adjacent subunits in the dimer complex of AMPA receptors at the level of the ligand-binding domain (LBD) near the first transmembrane domain [13–18]. Therefore, critical chimera with exchange of those putative binding site was first selected and compared to other chimera with exchange at N-terminal domain (NTD) that should be insensitive to AMPA positive modulators. Among GluA subunits, GluA1flop subtype was chosen as being markedly potentiated previously by exposure to S 47445. The selectivity of S 47445 towards AMPA receptors was first confirmed on human receptors, since S 47445 up to 100 μM did not modify glutamate-evoked current on human GluK2 kainate receptors (n = 12, Fig 8), as previously observed on rat kainate receptors. The potentiation of glutamate-evoked current persisted also after S 47445 application on AMPA/Kainate chimera GluA1(K2NTD), with exchange of amino-acid segment at the N-terminal portion (Fig 8) with an EC_50_ similar to those obtained on GluA1flop subtype (4.2 μM [1.3; 13.6], n = 5). In contrast, the effect of S 47445 observed on GluR1flop AMPA receptors was lost in AMPA/Kainate chimerae GluA1(K2S1), with exchange of the amino-acid segment corresponding to the putative binding site of AMPA positive modulators (n = 5, Fig 8). These results confirmed that the binding pocket located on the ligand-binding domain (LBD) near the first transmembrane domain is essential for inducing the modulation of glutamate-enhanced current by S 47445 as similarly observed for other AMPA positive modulators.

### Absence of toxicity of S 47445 on rat primary cortical cell cultures

**Fig 9:**
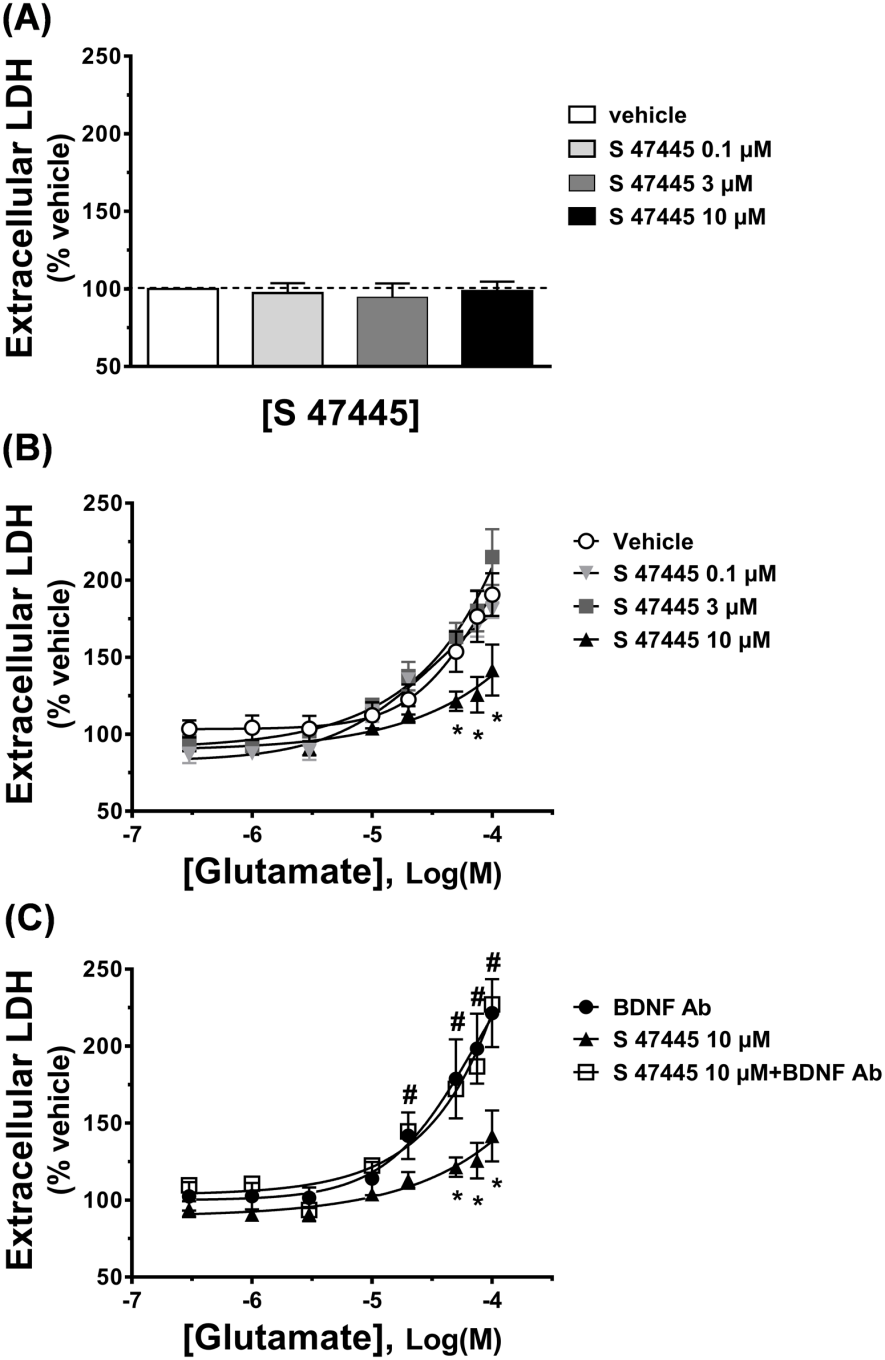
Effect of S 47445 on cellular toxicity on rat primary cortical cell cultures. Cell mortality was estimated by measurement of lactate deshydrogenase (LDH) and was normalized as a percentage of the one measured with vehicle. (A) Intrinsic toxicity induced by S 47445 at 0.1, 3 and 10 μM (n = 4 independent cultures), (B) Rat primary cortical neurons were exposed to a concentration response of glutamate (0.03, 1, 3, 10, 20, 50, 75 and 100 μM) for 10 min ± S 47445 (0.1, 3 and 10 μM) (n = 4 independent cultures). * p≤0.05 S 47445 10 μM compared to vehicle group, ANOVA followed by Fisher’s Protected Least Significant Difference and (C) Effect of S 47445 (10 μM) in presence of a neutralizing BDNF antibody (BDNF Ab) on glutamate-induced neuronal damage. * p≤0.05S 47445 10 μM compared to vehicle group; # p≤0.05 BDNF Ab/S 47445 10 μM compared to S 47445 10 μM group.

Aberrant or excessive activation of ionotropic glutamatergic receptors can result under some circumstances to cell death due to a phenomenon of excitotoxicity [2, 51]. Despite AMPA receptors are less involved in this phenomenon compared to NMDA [52] and kainate receptors and a positive modulation may avoid the attendant excitotoxicity effects of direct agonists, concerns over potential toxicity have been issued with AMPA-PAMs. Therefore, to evaluate the potential neurotoxic effect of the compound *in vitro*, S 47445 was incubated in absence or in presence of glutamate on cultured rat primary cortical neurones. S 47445 was not toxic to rat primary cortical cell cultures up to 10 μM after 24 hrs exposure when applied alone (n = 4 independent cultures, Fig 9A). Furthermore, in contrast to what may be expected following a potentiation of AMPA receptors, S 47445 (0.1, 3 and 10 μM) also did not potentiate glutamate-mediated toxicity on rat primary cortical neuronal culture, whatever the concentration of glutamate used (n = 4 independent cultures, Fig 9B). Instead, S 47445 at 10 μM elicited a significant protective effect against glutamate injury observed at 50, 75 and 100 μM by reducing glutamate-induced LDH activity by 32.2, 50.9 and 49.0% (Fig 9B). For comparison and as expected, glutamate at 50, 75 and 100 μM induced 54, 77 and 91% increase in LDH activity respectively, showing that glutamate at such concentration induced a significant increase in cell death (Fig 9B); further the NMDA antagonist MK801 (1 μM) completely reversed the 100 μM glutamate-mediated toxicity (data not shown).

A last series of experiments was set up in order to determine the underlying mechanism of the neuroprotective effect of S 47445. Several studies had linked the neuroprotective effects of AMPA-PAMs to stimulation of BDNF release [29, 37–39]. Based on these studies, a neutralizing anti-BDNF antibody was used in coapplication with S 47445 and glutamate on primary cortical neuronal cultures. The neuroprotective effect of S 47445 observed at 10 μM was completely blocked by the neutralizing BDNF antibody, confirming the involvement of this neurotrophin (Fig 9C).

### Effect of S 47445 on memory-like tests

AMPA-PAMs were reported to facilitate episodic and spatial working memories in a number of behavioral studies in rodents [15, 16, 20, 26, 29]. In order to further investigate the activity of S 47445, two screening cognitive tests were performed, one assessing episodic memory and the second one evaluating spatial working memory performance [33].

**Fig 10:**
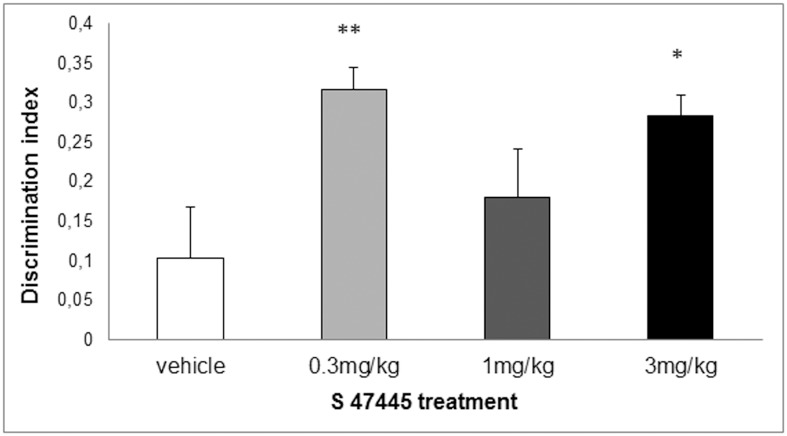
Effect of S 47445 in a novel object recognition task in rats (n = 10–11). Rats were treated p.o. either with S 47445 (0.3, 1 and 3 mg/kg) or vehicle, daily for 3 days i.e. 60 min before habituation, familiarisation and recognition phases. Histograms represent the mean ± SEM of the difference of the discrimination index during the recognition phase. * p≤0.05 and ** p≤0.01 versus vehicle group, one way ANOVA followed by a Dunnett’s test.

In the object recognition test in rats, post hoc Dunnett’s test performed following significant ANOVA showed that 3 administrations of S 47445 at 0.3 or 3 mg/kg p.o. significantly increased the discrimination index as compared to vehicle-treated rats (p ≤ 0.01 and p ≤ 0.05, respectively) whereas the global exploration was equivalent in the 4 groups (F(3,41) = 0.69). The tendency of increase observed after 1 mg/kg p.o. did not reach statistical significance (Fig 10). Exploration behaviour during T2 sessions was not altered by the treatment (data not shown).

**Fig 11:**
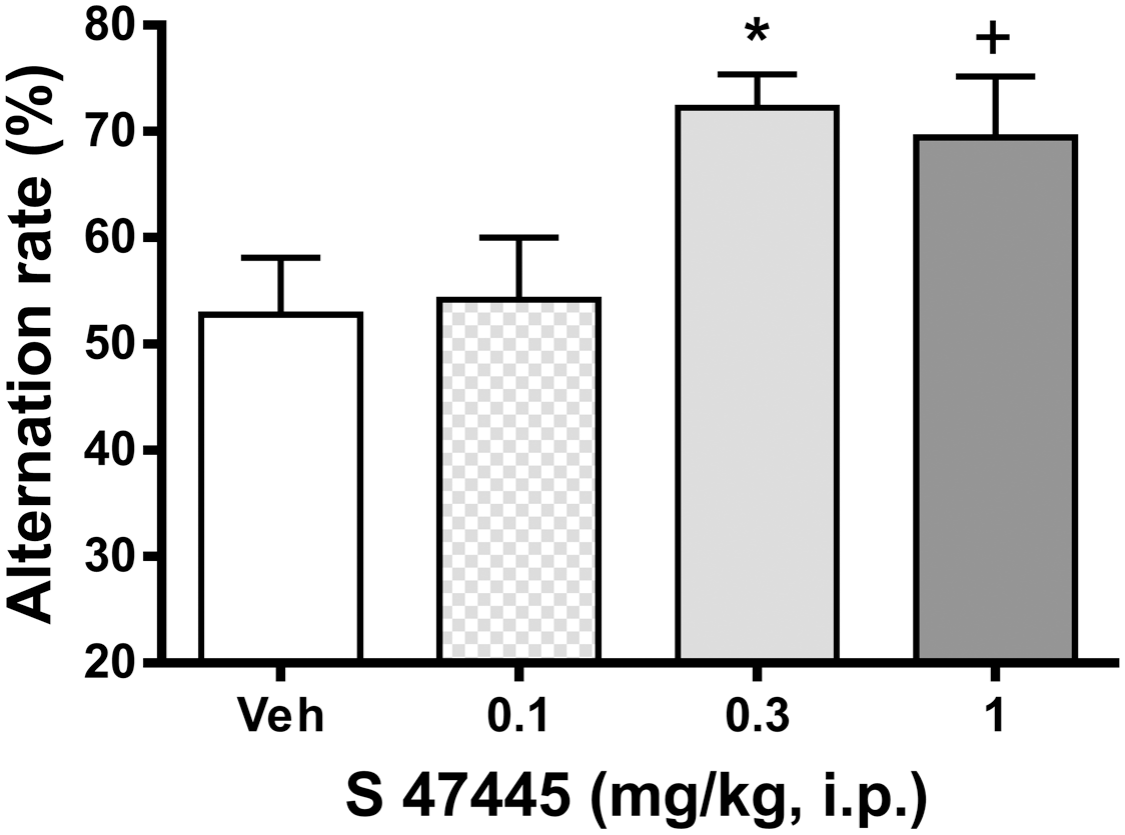
Effect of S 47445 on spontaneous alternation in T-maze in mice (n = 12). Mice were treated i.p. with either S 47445 (0.1, 0.3 and 1 mg/kg) or vehicle, 30 min prior to the test. Histograms illustrate the mean ± SEM of the percentage of spontaneous alternation of mice. ^+^ p = 0.07 and * p≤0.05 versus vehicle group, one way ANOVA followed by a Dunnett’s test.

In the alternation test in a T-maze, in which a long inter-trial delay (180 s) induced an impairment of the percentage of alternation in young mice, a one-way ANOVA showed a significant difference between groups (p≤0.05) over the 7 first trials. Post hoc Dunnett analysis showed that intraperitoneal acute administration of S 47445 at the doses of 0.3 and 1 mg/kg i.p. improved the performance of spontaneous alternation compared to vehicle-treated mice (p≤0.05 and p = 0.07 *versus* vehicle, respectively) (Fig 11). The global latency to enter into the arms was not affected by any doses of the treatment (data not shown).

### CNS safety in rodents

**Fig 12:**
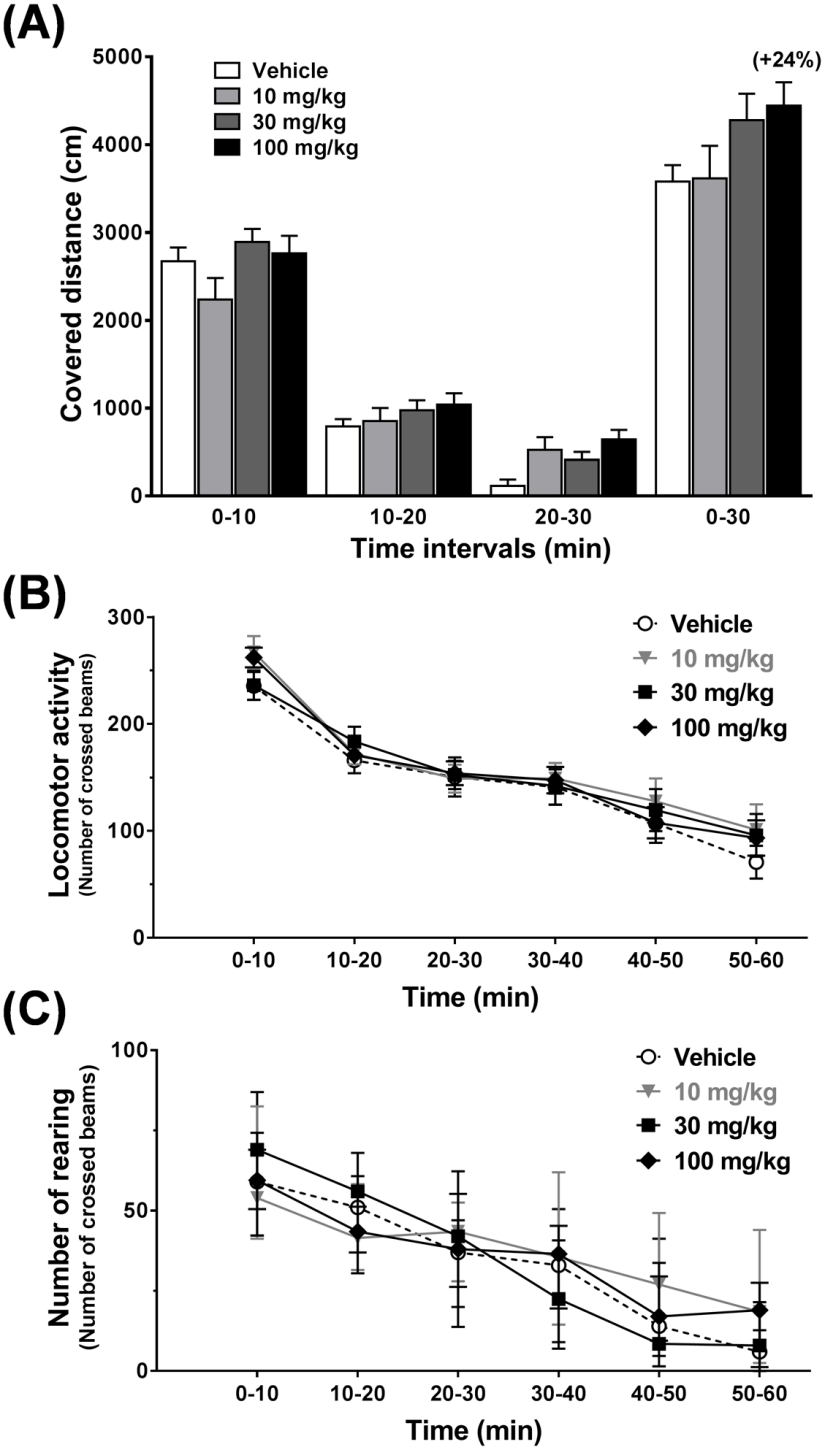
Effect of S 47445 on spontaneous locomotor activity in rats and mice. (A) Effect of S 47445 (10, 30 and 100 mg/kg, p.o.) on spontaneous locomotor activity recorded for 30 min, 1 hour after acute administration in Wistar rats (n = 9). Covered distance (cm) was expressed as means + SEM over 3 successive periods of 10 min (0–10, 10–20 and 20–30 min) and overall (0–30 min). Result indicated in brackets represents the percentage of variation as compared to vehicle. (B and C) Effect of S 47445 (10, 30 and 100 mg/kg, p.o.) on spontaneous locomotor activity recorded for 30 min, 1 hour after acute administration in NMRI mice (n = 12). Global activity (B) was expressed as mean ± SEM and number of rearing (C) as median and interquartile range over 6 successive periods of 10 min (0–10, 10–20, 20–30, 30–40, 40–50 and 50–60 min).

Acute administration of S 47445 (10, 30 and 100 mg/kg, p.o.) did not significantly modify the total distance of spontaneous locomotor activity in the rat (n = 9, Fig 12A) or the global spontaneous locomotor activity in NMRI mice (n = 9, Fig 12B). Also, it did not influence the number of rearing in mice (Fig 12C). Altogether these data confirmed the lack of effect of S 47445 up to 100 mg/kg on spontaneous locomotor activity in rats and mice. Moreover, oral acute administration of S 47445 had no significant effect on body temperature and behavior in mice (at 30, 100 and 300 mg/kg) and in rats (at 50, 200, 500 and 1000 mg/kg). No occurrence of spontaneous epileptic seizures was observed in mice and rats up to the highest tested dose (300 and 1000 mg/kg p.o., respectively).

## Discussion

Results presented herein provide evidence that S 47445 is a potent and selective AMPA-PAM since S 47445 has no affinity for orthosteric binding sites or agonist effects at AMPA receptors and its activity requires agonist activation of the receptor. In addition, S 47445 is deprived of activity at other glutamate ionotropic receptors (NMDA and kainate receptors) and did not show any binding affinity for more than 100 other ionotropic or G protein-coupled receptors, ion channels, monoamine transporters or enzymes, thus indicating no agonist or antagonist activities on these different targets as no displacement of the respective ligands were clearly observed.

Using electrophysiological recordings on *Xenopus laevis* oocytes or HEK-293 cells, S 47445 enhanced in a concentration-dependent manner the agonist-evoked responses, with no effect when applied alone. S 47445 potentiated glutamate-evoked currents by slowing the rate of onset of desentitization and by increasing the apparent affinity of glutamate. S 47445 presented potent cognitive-enhancing and neurotrophic properties *in vitro*. Nevertheless, as glutamate enhancer, S 47445 did not evoke neurotoxic effect *in vitro* or induced epileptic seizures at high doses. In contrast, S 47445 presented *in vitro* neuroprotective effects in relation with its neurotrophic properties.

Comparing S 47445 activity with other AMPA-PAMs, S 47445 was found to be more potent than the benzothiadiazine S 18986 and the benzamide CX614, less potent than the biaryl propylsulfonamide LY451395, and equivalent to the benzothiadiazine cyclothiazide and biaryl propylsulfonamide LY404187. Besides, the maximal magnitude of potentiation of AMPA-evoked currents is less important with S 47445 than with other AMPA-PAMs, an observation that may account for the absence of neurotoxicity of S 47445 observed *in vitro*. In HEK-293 cells expressing human GluA1flop/GluA2flip AMPA receptors subtypes, coapplication of 0.1 μM of S 47445 with glutamate caused a marked increase of the glutamate responses and an increase of the apparent affinity for glutamate with a leftward shift in the EC_50_ of about 16 fold, as described for typical allosteric modulation [50]. For comparison, approximately 7-fold increase of agonist potency was previously described with LY392098 on isolated prefrontal cortex pyramidal neurons using patch-clamp technique or with the biaryl propylsulfonamide PEPA on GluA3flop receptors expressed in *Xenopus* oocytes [24, 53]. Additionally, by using repetitive glutamate pulses on human GluA1flop/GluA2flip AMPA receptors subtypes expressed in *Xenopus* oocytes, we showed that low concentrations of S 47445 (0.1 and 0.3 μM) produced a progressive potentiation of the glutamate-evoked currents starting from the second pulse of glutamate not accompanied by the development of desensitization following several pulses of the agonist. This suggests that S 47445 is characterised both by a priming effect on the glutamate-evoked response with a potentiation of this response observed at concentrations of the drug well below the EC_50_, and also by an absence of desensitization of the drug effect in this experimental model. We further confirmed the positive modulation of S 47445 on AMPA receptors subtypes since the potentiation effect of S 47445 (1 μM) on the amplitude of the glutamate-evoked response was concentration-dependently reversed by the selective non-competitive AMPA receptor antagonist GYKI52466 [54, 55] in *Xenopus* oocytes. As exposure to GYKI52466 caused only a reduction of the amplitude of the S 47445 potentiation but no shift in sensitivity, this indicates that S 47445 and GYKI52466 are acting on separate binding sites of AMPA receptors, as previously shown for cyclothiazide and CX614 [54, 56, 57]. The lack of competitive interaction of AMPA-PAMs and GYKI52466 was also confirmed by the fact that GYKI52466 binds to small regions of the interface between the S1 and S2 glutamate binding core and channel transmembrane domains M1 and M4, at S1-M1 and S2-M4 linkers [58], close but different from the common binding pocket identified for AMPA-PAMs [13–18]. These results are in agreement with work done by Yamada and Turetsky showing that cyclothiazide exerts allosteric modulatory effects upon two sites with both competitive and non-competitive mechanism of action [57]. We also demonstrated using AMPA/kainate chimera approach that allosteric modulation of S 47445 involved binding at the binding pocket of AMPA-PAMs located at the S1 segment within the dimer interface of the LBD of AMPA receptors. Indeed, S 47445 potentiated effect was lost at both GluK2 kainate receptors and GluA1(K2S1) chimera, with exchange of amino-acid at the S1 segment between GluA1flop and GluK2 subunits, but persisted in GluA1(K2NTD) chimera, with exchange of amino-acid segment at the N-Terminal. This result is in line with the binding pocket described for various AMPA-PAMs located on the ligand-binding domain (LBD) near the first transmembrane domain [13–18]. More precise mapping of the binding site is expected to be obtained using site directed mutagenesis. Nevertheless, interaction of other AMPA-PAMs with the receptor differs in several way and mechanisms. Pharmacology of AMPA-PAMs depends on the occupancy of selective subsites of the LBD (one or two compounds per binding pocket, interaction with central, hydrophobic and/or hydrophilic sites), on modulation of desensitization and/or deactivation of the receptors, on different subunit selectivity and splice variant sensitivity. For instance, aniracetam had little effect on channel desensitization, cyclothiazide and LY404187 had little effect on channel deactivation and CX614 and JAMI1001A unexpectedly modulated both desensitization and deactivation of the receptors [13, 18, 59]. The AMPA receptors modulation by low concentration of S 47445 was accompanied by a strong reduction of the decay time of the response. Selectivity of S 47445 for human AMPA receptor subtypes was assessed using GluA1, GluA2 and GluA4 subunits and their splice variants expressed in oocytes. S 47445 concentration-dependently potentiated glutamate-evoked currents in oocytes with similar EC_50_ among most homomeric and heteromeric GluA recombinants, except at GluA4 flop subtype suggesting a moderate discrimination of S 47445 between subtypes of AMPA receptors. Additionally, the amount of potentiation of glutamate-evoked currents by S 47445 was consistency greater at flop than flip variants reaching significance for GluA1flop *versus* GluA1flip homomers and Glu1flop/2flip *versus* Glu1flip/2flip heteromers. Splice variant selectivity of others AMPA-PAMs differs from S 47445. Most of them target preferentially the flip isoform such as cyclothiazide [21, 23], LY404187 and LY503430 [22, 60, 61]. Few PAM-AMPAs such as CX614 [18] or PEPA [24, 62] showed a greater magnitude of potentiation for the flop isoforms with less potency effect at EC_50_. Here again, the flip/flop region seems critical since residues within this domain are necessary for the effect of cyclothiazide or LY404187 against AMPA desensitization [61, 63, 64]. The target of some isoforms present in specific brain regions may represent an advantage for the development of selective drugs with stronger efficacy and less side effects. Interestingly, flop variants are associated with faster desensitization rate and more rapid closing of AMPA receptors following activation than flip variants [11, 12]. It can be speculated that such flop preference as observed for S 47445 on GluA1 containing receptors may help to limit neurotoxic effects associated with the duration of potentiation of glutamatergic transmission.

A series of experiments were dedicated to investigate S 47445 potential neurotoxic effect due to risk of glutamate excitotoxicity enhancement. AMPA-PAMs may have advantage over the use of agonists regarding excitotoxicity, as AMPA-PAMs are agonist-dependent, increase the response or lower the activation threshold but leave the natural neurotransmission intact. S 47445 alone for 24h showed no toxicity up to 10 μM on rat primary cortical brain cells and did not potentiate glutamate-mediated toxicity as well. In contrast, a neuroprotection against glutamate-toxicity was noticed at 10μM. This neuroprotective effect involved an increase expression or release of BDNF since it was totally reversed by co-administration of neutralizing anti-BDNF antibody. In line with this, S 47445 demonstrated already neurotrophic properties by inducing mature form of BDNF proteins in aged rats from the dose of 1 mg/kg [63]. BDNF modulates synaptic connectivity especially by sculpting postsynaptic spines and regulates synaptic transmission thereby promoting synaptic plasticity and related memory processes [36, 65]. A variety of neurotrophic factors, including BDNF, have been reported to promote the survivals of neurons in culture and to protect against excitotoxic or neurotoxin-induced lesions in animal models of stroke or Parkinson’s disease [66]. Similarly, significant neuroprotective effect against excitotoxic ibotenate-induced lesions on both cortical plate and white matter were previously observed with other AMPA-PAMs [37, 67]. Other lines of work indicated that neuroprotective effects of AMPA-PAMs in Parkinson or Huntington animal models may be mediated by increased BDNF expression [15, 38, 68].

The present results provide evidence that S 47445 from 0.3 mg/kg per os displayed procognitive properties in classical memory-like adult rodent models of both episodic and working memory. Moreover, the procognitive effects of S 47445 are unlikely to be due to nonspecific CNS effects since S 47445 showed no deleterious effects on locomotor activity. These observations on memory are in excellent agreement with the body of evidence demonstrating that AMPA-PAMs facilitate cognitive function in a variety of species ranging from rodents to humans [15, 16, 20, 26, 29, 30]. Further, it was observed that S 47445 given acutely at 10 mg/kg p.o. significantly increased long term potentiation (LTP) in CA3-CA1 hippocampal synapses in young alert mice as compaired to vehicle mice [69]. Furthermore, chronic treatment with S 47445 at 10 mg/kg in old alert mice significantly counteracted the deficit of LTP due to age at the CA3-CA1 synapse level [69]. These *in vivo* effects of S 47445 on hippocampal neuroplasticity also support its memory-enhancing properties observed herein.

We note from previous kinetic experiments that the cerebral C_max_ obtained after oral administration at the first active dose 0.3 mg/kg in rats and mice was around 0.1–0.2 μM. Interestingly, we presently demonstrated on both HEK-293 cells and on oocytes expressing human GluA1flop/GluA2flip AMPA receptors subtypes, that S 47445 from the low concentration of 0.1 μM was indeed able to induce a potent, rapid and sustained potentiation of glutamate-evoked currents. These findings suggest that a low concentration of S 47445 in the brain is sufficient to produce a positive modulation of AMPA receptors and to trigger procognitive effects. Finally, S 47445 was also very well-tolerated in rodents, as no adverse effect or occurrence of spontaneous epileptic seizures was noticed after oral acute administration of high doses up to 300 and 1000 mg/kg in mice and rats, respectively.

## Conclusions

In conclusion, S 47445 is a potent and selective AMPA-PAM with a larger magnitude of potentiation at the flop splice isoform of GluA1 AMPA receptor subunits. S 47445 displays some cognitive-enhancing properties in preclinical memory-like models and presents both potential neurotrophic and neuroprotective effects *in vitro*. Therefore, S 47445 is expected to provide beneficial effects in diseases associated with glutamatergic disorders such as Major Depressive Disorder or Alzheimer’s disease. Two phase II studies are currently on-going in these 2 populations.

## Supporting information

S1 Table Competition binding assays and enzyme and cell-based assays experiments. Affinities of S 47445 were evaluated in duplicate at two concentrations (0.1 and 10 μM) on the following panel of receptors, channels, enzymes and transporters.
(DOCX)
