# Supplementary material for: Pharmacological characterisation of S 47445, a novel positive allosteric modulator of AMPA receptors
Source: PLoS One. 2017 Sep 8;12(9):e0184429. doi: 10.1371/journal.pone.0184429 (PMC5590943; doi:10.1371/journal.pone.0184429)
Supplement: S1 Table — Affinities of S 47445 were evaluated in duplicate at two concentrations (0.1 and 10 μM) on the following panel of receptors, channels, enzymes and transporters. (DOCX) [file pone.0184429.s001.docx]

**Supporting information**

**Supplementary Table 1. Competition binding assays and enzyme and cell-based assays experiments**.

| Binding assay | Ligand | S 47445 Concentration (M) | % Inhibition of control specific binding |
| --- | --- | --- | --- |
| A_1_ (h) | [^3^H]DPCPX | 1.0E-07 | 8 |
|  |  | 1.0E-05 | 35 |
| A_2A_ (h) | [^3^H]CGS 21680 | 1.0E-07 | -8 |
|  |  | 1.0E-05 | 9 |
| α1 (non-selective) | [^3^H]prazosin | 1.0E-07 | -5 |
|  |  | 1.0E-05 | 1 |
| α1A | [^3^H]prazosin | 1.0E-07 | 4 |
|  |  | 1.0E-05 | -2 |
| α1B (h) | [^3^H]prazosin | 1.0E-07 | 8 |
|  |  | 1.0E-05 | 19 |
| α1D (h) | [^3^H]prazosin | 1.0E-07 | 0 |
|  |  | 1.0E-05 | 8 |
| α2 (non-selective) | [^3^H]RX 821002 | 1.0E-07 | -2 |
|  |  | 1.0E-05 | -5 |
| α2A (h) | [^3^H]RX 821002 | 1.0E-07 | 4 |
|  |  | 1.0E-05 | 2 |
| α2B (h) | [^3^H]RX 821002 | 1.0E-07 | -9 |
|  |  | 1.0E-05 | -6 |
| α2C (h) | [^3^H]RX 821002 | 1.0E-07 | 8 |
|  |  | 1.0E-05 | 4 |
| β1 (h) | [^3^H](-)CGP 12177 | 1.0E-07 | -1 |
|  |  | 1.0E-05 | 1 |
| β2 (h) | [^3^H](-)CGP 12177 | 1.0E-07 | -3 |
|  |  | 1.0E-05 | 2 |
| AT1 (h) | [^125^I][Sar^1^,Ile^8^]-AT II | 1.0E-07 | -1 |
|  |  | 1.0E-05 | 3 |
| BZD (central) | [^3^H]flunitrazepam | 1.0E-07 | -2 |
|  |  | 1.0E-05 | -21 |
| B_2_ (h) | [^3^H]bradykinin | 1.0E-07 | 3 |
|  |  | 1.0E-05 | 2 |
| CGRP (h) | [^125^I]hCGRPα | 1.0E-07 | -12 |
|  |  | 1.0E-05 | 22 |
| CB_1_ (h) | [^3^H]CP 55940 | 1.0E-07 | 2 |
|  |  | 1.0E-05 | 18 |
| CB_2_ (h) | [^3^H]WIN 55212-2 | 1.0E-07 | 8 |
|  |  | 1.0E-05 | 15 |
| CCK_A_ (h) (CCK1) | [^125^I]CCK-8s | 1.0E-07 | 4 |
|  |  | 1.0E-05 | -15 |
| D_1_ (h) | [^3^H]SCH 23390 | 1.0E-07 | 10 |
|  |  | 1.0E-05 | -4 |
| D_2S_ (h) | [^3^H]spiperone | 1.0E-07 | -2 |
|  |  | 1.0E-05 | -2 |
| D_3_ (h) | [^3^H]spiperone | 1.0E-07 | 5 |
|  |  | 1.0E-05 | -3 |

| Binding assay | Ligand | S 47445 Concentration (M) | % Inhibition of control specific binding |
| --- | --- | --- | --- |
| D_4.4_ (h) | [^3^H]spiperone | 1.0E-07 | 2 |
|  |  | 1.0E-05 | 10 |
| D_5_ (h) | [^3^H]SCH 23390 | 1.0E-07 | -1 |
|  |  | 1.0E-05 | -8 |
| ET_A_ (h) | [^125^I]endothelin-1 | 1.0E-07 | -15 |
|  |  | 1.0E-05 | -11 |
| ET_B_ (h) | [^125^I]endothelin-1 | 1.0E-07 | -4 |
|  |  | 1.0E-05 | -3 |
| GABA (non-selective) | [^3^H]GABA | 1.0E-07 | 4 |
|  |  | 1.0E-05 | 7 |
| AMPA | [^3^H]AMPA | 1.0E-07 | 1 |
|  |  | 1.0E-05 | -8 |
| Kainate | [^3^H]kainic acid | 1.0E-07 | 7 |
|  |  | 1.0E-05 | 25 |
| NMDA | [^3^H]CGP 39653 | 1.0E-07 | 2 |
|  |  | 1.0E-05 | -1 |
| H_1_ (h) | [^3^H]pyrilamine | 1.0E-07 | 2 |
|  |  | 1.0E-05 | 8 |
| H_2_ (h) | [^125^I]APT | 1.0E-07 | -13 |
|  |  | 1.0E-05 | 5 |
| H_3_ (h) | [^3^H]N^α^-Me-histamine | 1.0E-07 | -6 |
|  |  | 1.0E-05 | 4 |
| H_4_ (h) | [^3^H]histamine | 1.0E-07 | -1 |
|  |  | 1.0E-05 | -2 |
| I_1_ | [^3^H]clonidine (+ 10 μM RX821002) | 1.0E-07 | 10 |
|  |  | 1.0E-05 | 21 |
| I_2_ | [^3^H]idazoxan | 1.0E-07 | -5 |
|  |  | 1.0E-05 | 29 |
| LTB_4_ (h) (BLT1) | [^3^H]LTB_4_ | 1.0E-07 | -4 |
|  |  | 1.0E-05 | 6 |
| MT_1_ (h) | [^125^I]2-iodomelatonin | 1.0E-07 | 1 |
|  |  | 1.0E-05 | 9 |
| MT2 (h) | [^125^I]2-iodomelatonin | 1.0E-07 | 1 |
|  |  | 1.0E-05 | 1 |
| M_1_ (h) | [^3^H]pirenzepine | 1.0E-07 | -5 |
|  |  | 1.0E-05 | 7 |
| M_2_ (h) | [^3^H]AF-DX 384 | 1.0E-07 | -1 |
|  |  | 1.0E-05 | 2 |
| M_3_ (h) | [^3^H]4-DAMP | 1.0E-07 | 0 |
|  |  | 1.0E-05 | 3 |
| M_4_ (h) | [^3^H]4-DAMP | 1.0E-07 | -3 |
|  |  | 1.0E-05 | -2 |
| NK_1_ (h) | [^125^I]BH-SP | 1.0E-07 | -9 |
|  |  | 1.0E-05 | -5 |
| NK_2_ (h) | [^125^I]NKA | 1.0E-07 | -2 |
|  |  | 1.0E-05 | 4 |
| Y (non-selective) | [^3^H]NPY | 1.0E-07 | -4 |
|  |  | 1.0E-05 | -6 |
| N (neuronal) (a-BGTX-insensitive) (a4b2) | [^3^H]cytisine | 1.0E-07 | 13 |
|  |  | 1.0E-05 | 6 |
| N (neuronal) (a-BGTX-sensitive) (a7) | [^125^I]α-bungarotoxin | 1.0E-07 | -13 |
|  |  | 1.0E-05 | -8 |

| Binding assay | Ligand | S 47445 Concentration (M) | % Inhibition of control specific binding |
| --- | --- | --- | --- |
| d_2_ (h) (DOP) | [^3^H]DADLE | 1.0E-07 | 2 |
|  |  | 1.0E-05 | 0 |
| k (KOP) | [^3^H]U 69593 | 1.0E-07 | -1 |
|  |  | 1.0E-05 | 6 |
| m (h) (MOP) (agonist site) | [^3^H]DAMGO | 1.0E-07 | -7 |
|  |  | 1.0E-05 | -4 |
| PPARg (h) | [^3^H]rosiglitazone | 1.0E-07 | 11 |
|  |  | 1.0E-05 | 17 |
| TP (h) (TXA2/PGH2) | [^3^H]SQ 29548 | 1.0E-07 | -6 |
|  |  | 1.0E-05 | -11 |
| 5-HT_1A_ (h) | [^3^H]8-OH-DPAT | 1.0E-07 | 5 |
|  |  | 1.0E-05 | 0 |
| 5-HT_1B_ | [^125^I]CYP (+ 30 μM (-)propranolol) | 1.0E-07 | -7 |
|  |  | 1.0E-05 | -3 |
| 5-HT_1D_ | [^3^H]serotonin | 1.0E-07 | -1 |
|  |  | 1.0E-05 | -3 |
| 5-HT_2A_ (h) | [^3^H]ketanserin | 1.0E-07 | -1 |
|  |  | 1.0E-05 | 5 |
| 5-HT_2B_ (h) (agonist site) | [^125^I](±)DOI | 1.0E-07 | 4 |
|  |  | 1.0E-05 | 10 |
| 5-HT_2C_ (h) | [^3^H]mesulergine | 1.0E-07 | -5 |
|  |  | 1.0E-05 | 2 |
| 5-HT_3_ (h) | [^3^H]BRL 43694 | 1.0E-07 | 2 |
|  |  | 1.0E-05 | 7 |
| 5-HT_4e_ (h) | [^3^H]GR 113808 | 1.0E-07 | 1 |
|  |  | 1.0E-05 | 0 |
| s1 (h) | [^3^H](+)pentazocine | 1.0E-07 | -15 |
|  |  | 1.0E-05 | 10 |
| s2 | [^3^H]DTG (+ 300 nM (+)pentazocine) | 1.0E-07 | 9 |
|  |  | 1.0E-05 | 30 |
| Estrogen a (h) (ERa) | fluormone^TM^ES2 | 1.0E-07 | 4 |
|  |  | 1.0E-05 | 5 |
| Progesterone (h) (PR) | [^3^H]R 5020 | 1.0E-07 | 22 |
|  |  | 1.0E-05 | 20 |
| Androgen (h) (AR) | [^3^H]methyltrienolone | 1.0E-07 | 0 |
|  |  | 1.0E-05 | -5 |
| Ca2+ channel  (L, DHP site) | [^3^H](+)PN 200-110 | 1.0E-07 | -16 |
|  |  | 1.0E-05 | 1 |
| Ca2+ channel (L, diltiazem site)  (benzothiazepines) | [^3^H]diltiazem | 1.0E-07 | -6 |
|  |  | 1.0E-05 | 25 |
| Ca2+ channel (L, verapamil site)  phenylalkylamines) | [^3^H](-)D 888 | 1.0E-07 | 4 |
|  |  | 1.0E-05 | -1 |
| K^+^_ATP_ channel | [^3^H]glibenclamide | 1.0E-07 | 6 |
|  |  | 1.0E-05 | 0 |
| K^+^_V_ channel | [^125^I]α−dendrotoxin | 1.0E-07 | -6 |
|  |  | 1.0E-05 | -2 |
| SK^+^_Ca_ channel | [^125^I]apamin | 1.0E-07 | 13 |
|  |  | 1.0E-05 | -10 |

| Binding assay | Ligand | S 47445 Concentration (M) | % Inhibition of control specific binding |
| --- | --- | --- | --- |
| Na+ channel (site 2) | [^3^H]batrachotoxinin | 1.0E-07 | 2 |
|  |  | 1.0E-05 | 41 |
| NE transporter (h) | [^3^H]nisoxetine | 1.0E-07 | 2 |
|  |  | 1.0E-05 | -1 |
| DA transporter (h) | [^3^H]BTCP | 1.0E-07 | 10 |
|  |  | 1.0E-05 | 19 |
| Choline transporter (h) (CHT1) | [^3^H]hemicholinium-3 | 1.0E-07 | -1 |
|  |  | 1.0E-05 | -1 |
| 5-HT transporter (h) | [^3^H]imipramine | 1.0E-07 | -6 |
|  |  | 1.0E-05 | 0 |

| Enzyme and  Cell-based Assay | | Ligand | S 47445 Concentration (M) | | % Inhibition of control specific binding | |
| --- | --- | --- | --- | --- | --- | --- |
| Phospholipase A_2_ | | [^3^H]phosphatidylethanolamine | 1.0E-07 | | 0 | |
|  |  |  | 1.0E-05 | | -4 | |
| COX_1_ (h) | | arachidonic acid | 1.0E-07 | | 8 | |
|  |  | (4 μM) | 1.0E-05 | | 9 | |
| COX_2_ (h) | | arachidonic acid | 1.0E-07 | | -1 | |
|  |  | (2 μM) | 1.0E-05 | | -8 | |
| 12-LO (h) | | arachidonic acid | 1.0E-07 | | -1 | |
|  |  | (6 μM) | 1.0E-05 | | -4 | |
| 15-LO (soybean) | | arachidonic acid | 1.0E-07 | | -2 | |
|  |  | (35 μM) | 1.0E-05 | | 2 | |
| inducible NOS (isol. enz/ spectrophoto.) | | arginine | 1.0E-07 | | 2 | |
|  |  | (100 μM) | 1.0E-05 | | 2 | |
| constitutive NOS (h) (endothelial) | | [^3^H]arginine (28 nM) | 1.0E-07 | | 2 | |
|  |  | + arginine (50 nM) | 1.0E-05 | | 1 | |
| PDE1 | | [^3^H]cAMP | 1.0E-07 | | 1 | |
|  |  | + cAMP (1 μM) | 1.0E-05 | | 10 | |
| PDE2 (h) | | [^3^H]cAMP | 1.0E-07 | | -4 | |
|  |  | + cAMP (1 μM) | 1.0E-05 | | -3 | |
| PDE3 (h) | | [^3^H]cAMP + cAMP (0.1 μM) | 1.0E-07 | | 0 | |
|  |  |  | 1.0E-05 | | -5 | |
| PDE4 (h) | | [^3^H]cAMP | 1.0E-07 | | 20 | |
|  |  | + cAMP (1 μM) | 1.0E-05 | | -12 | |
| PDE5 (h) | | [^3^H]cGMP + cGMP (1 μM) | 1.0E-07 | | -9 | |
|  |  |  | 1.0E-05 | | -11 | |
| ACE (h) | | Mca-Arg-Pro-Pro-Gly-Phe- Ser-Ala-Phe-Lys (DNP)-OH (10 μM) | 1.0E-07 | | 4 | |
|  |  |  | 1.0E-05 | | 8 | |
| ECE-1 (h) | | ECE-1 fluorescent substrate (15 μM) | 1.0E-07 | | 3 | |
|  |  |  | 1.0E-05 | | 3 | |
| Caspase-1 (h) | | benzyloxycarbonyl- Tyr-Val-Ala-Asp-AFC (10 μM) | 1.0E-07 | | -3 | |
|  |  |  | 1.0E-05 | | -5 | |
| Caspase-3 (h) | | benzyloxycarbonyl- Asp-Glu-Val-Asp-AFC (3.6 μM) | 1.0E-07 | | -19 | |
|  |  |  | 1.0E-05 | | -3 | |
| Caspase-8 (h) | benzyloxycarbonyl- Ile-Glu-Thr-Asp-AFC (10 μM) | | | 1.0E-07 | | 2 |
|  |  |  |  | 1.0E-05 | | -2 |

| Enzyme and  Cell-based Assay | Ligand | | S 47445 Concentration (M) | | % Inhibition of control specific binding |
| --- | --- | --- | --- | --- | --- |
| Adenylyl cyclase (stimulated) | ATP (0.5 mM) / | | 1.0E-07 | | 6 |
|  | forskolin (10 μM) | | 1.0E-05 | | -21 |
| Guanylyl cyclase (stimulated) | GTP (0.1 mM) / | | 1.0E-07 | | -14 |
|  | SNP (10 μM) | | 1.0E-05 | | 7 |
| Abl kinase (h) | ATP + biotinyl- βAβAβAEAIYAAPFAKKK (0.2 μM) | | 1.0E-07 | | -4 |
|  |  |  | 1.0E-05 | | -5 |
| Akt1/PKBa (h) | ATP + biotinyl- βAβAβARARTSSFAEPG (0.03 μM) | | 1.0E-07 | | -7 |
|  |  |  | 1.0E-05 | | -7 |
| CaMK2a (h) | ATP + biotinyl- βAβAβAKKALRRQETVDAL (0.1 μM) | | 1.0E-07 | | 0 |
|  |  |  | 1.0E-05 | | -1 |
| CDC2/CDK1 (h) (cycB) | ATP + biotinyl-Rb-derived peptide (0.4 μM) | | 1.0E-07 | | 5 |
|  |  |  | 1.0E-05 | | 5 |
| EGFR kinase (h) | ATP + biotinyl- βAβAβAAEEEEYFELVAKKK (0.1 μM) | | 1.0E-07 | | -8 |
|  |  |  | 1.0E-05 | | 0 |
| FGFR1 kinase (h) | βAβAβAAAEEEYFFLFAKKK (150 nM) | | 1.0E-07 | | 8 |
|  |  |  | 1.0E-05 | | 0 |
| IRK (h) (InsR) | | ATP + biotinyl- βAβAβAAAEEEYMMMFAKKK | 1.0E-07 | | -2 |
|  |  |  | 1.0E-05 | | 1 |
| Lyn kinase (h) | | ATP + biotinyl- βAβAβAKVEKIGEGTYGVVYK (0.1 μM) | 1.0E-07 | | -1 |
|  |  |  | 1.0E-05 | | -2 |
| MEK1/MAP2K1 (h) | | ATP + inactivated ERK2 (0.1 μM) | 1.0E-07 | | -9 |
|  |  |  | 1.0E-05 | | -1 |
| p38a kinase (h) | | ATP + ATF-2 (0.1 μM) | 1.0E-07 | | 2 |
|  |  |  | 1.0E-05 | | 4 |
| PKA (h) | | ATP + biotinyl- CREB-derived peptide (10 nM) | 1.0E-07 | | 7 |
|  |  |  | 1.0E-05 | | 9 |
| PKCα (h) | | ATP + biotinylneurogranin 28-43 peptide (60 nM) | 1.0E-07 | | 1 |
|  |  |  | 1.0E-05 | | 8 |
| RAF-1 kinase (h) | | ATP + inactive MEK1 (0.2 μM) | 1.0E-07 | | 0 |
|  |  |  | 1.0E-05 | | -3 |
| TRKB (h) | | ATP + PLCγ biotinylated peptide (0.1 μM) | 1.0E-07 | | 0 |
|  |  |  | 1.0E-05 | | 3 |
| Phospholipase C | | [^3^H]PIP2 (0.3 mM) | 1.0E-07 | | -5 |
|  |  |  | 1.0E-05 | | 7 |
| Acetylcholinesterase (h) | | AMTCh | 1.0E-07 | 0 | |
|  |  | (50 μM) | 1.0E-05 | 21 | |
| MAO-A (h) | | kynuramine (0.15 mM) | 1.0E-07 | -3 | |
|  |  |  | 1.0E-05 | -2 | |
| MAO-B (h) | | benzylamine | 1.0E-07 | -1 | |
|  |  | (0.5 mM) | 1.0E-05 | 39 | |
| ATPase (Na+/K+) | | ATP | 1.0E-07 | -3 | |
|  |  | (2 mM) | 1.0E-05 | -2 | |

Affinities of S 47445 were evaluated in duplicate at two concentrations (0.1 and 10 µM) on the following panel of receptors, channels, enzymes and transporters.

Table with detailed data results are available in supplementary information.
